# *p*-Coumaroyl Amides from the Plant Kingdom: A Comprehensive Review of Natural Sources, Biosynthesis, and Biological Activities

**DOI:** 10.3390/molecules30061259

**Published:** 2025-03-11

**Authors:** Federico Berti, Elena Maria Tamburello, Cristina Forzato

**Affiliations:** Dipartimento di Scienze Chimiche e Farmaceutiche, Università degli Studi di Trieste, Via L. Giorgieri 1, 34127 Trieste, Italy; fberti@units.it (F.B.); elenamaria.tamburello@phd.units.it (E.M.T.)

**Keywords:** *p*-coumaric acid, amides, secondary metabolites, conjugates

## Abstract

Hydroxycinnamic acids are widely distributed in the plant kingdom, both as free compounds and as conjugates with other molecules, such as amino acids, carbohydrates, alcohols or amines, and polyamines, forming different derivatives, such as amides, esters, thioesters, or ethers. Among the different hydroxycinnamic acids, *p*-coumaric acid has a high bioavailability and its amide derivatives, also known as phenolamides (PAs) and hydroxycinnamic acid amides (HCAAs), play specific roles in plant development and defense. They are also involved in several biological activities that affect human health. The present review collected data and described secondary and tertiary amides of *p*-coumaric acids found in plants, from their natural sources to their biosynthesis. The review also described the acyl-transferase mechanisms involved in their formation, their roles in plants, as well as studies of their biological activities in humans.

## 1. Introduction

The production of secondary metabolites by plants, which are an expression of the individuality of species, can have different purposes for the organism, such as defense against predators by producing toxic materials, attracting other species by volatile compounds or coloring agents biosynthesis, or simply as waste products. Plants also produce specific secondary metabolites to resist biotic and abiotic stresses or as plant growth regulators. Phenolamides (PAs) are one of the largest classes of plant-specialized secondary metabolites. These compounds are found ubiquitously in plants and play an important role in a wide range of biological processes, such as plant development and defense [1,2]. Moreover, a large number of biological studies have recently shown that these compounds can have many beneficial functions in the human body, including anti-oxidant, anti-inflammatory, anti-cancer, anti-tyrosinase, and neuroprotective. In general, PAs are obtained from phenolic acids with aliphatic or aromatic amines and since these building blocks are widely present in nature, thousands of different compounds can be obtained.

Among the phenolic acids, hydroxycinnamic acids are one of the most abundant in the plant kingdom; they can be found as free hydroxycinnamic acids or as conjugates with other compounds such as chlorogenic acids, rosmarinic acids, or cinnamoyl amides [3,4].

*p*-Coumaric acid is one of the hydroxycinnamic acids found at significant levels in many fruits, vegetables, and cereals and can exert different beneficial effects on human health, e.g., inflammation, cardiovascular diseases, diabetes, and nervous system diseases [5].

The present review focuses on *p*-coumaroyl amide derivatives, giving an overview of all conjugates produced by plants, including their biosynthesis, their role in plants, and the effect of their biological activity on human health.

**Scheme 1:**
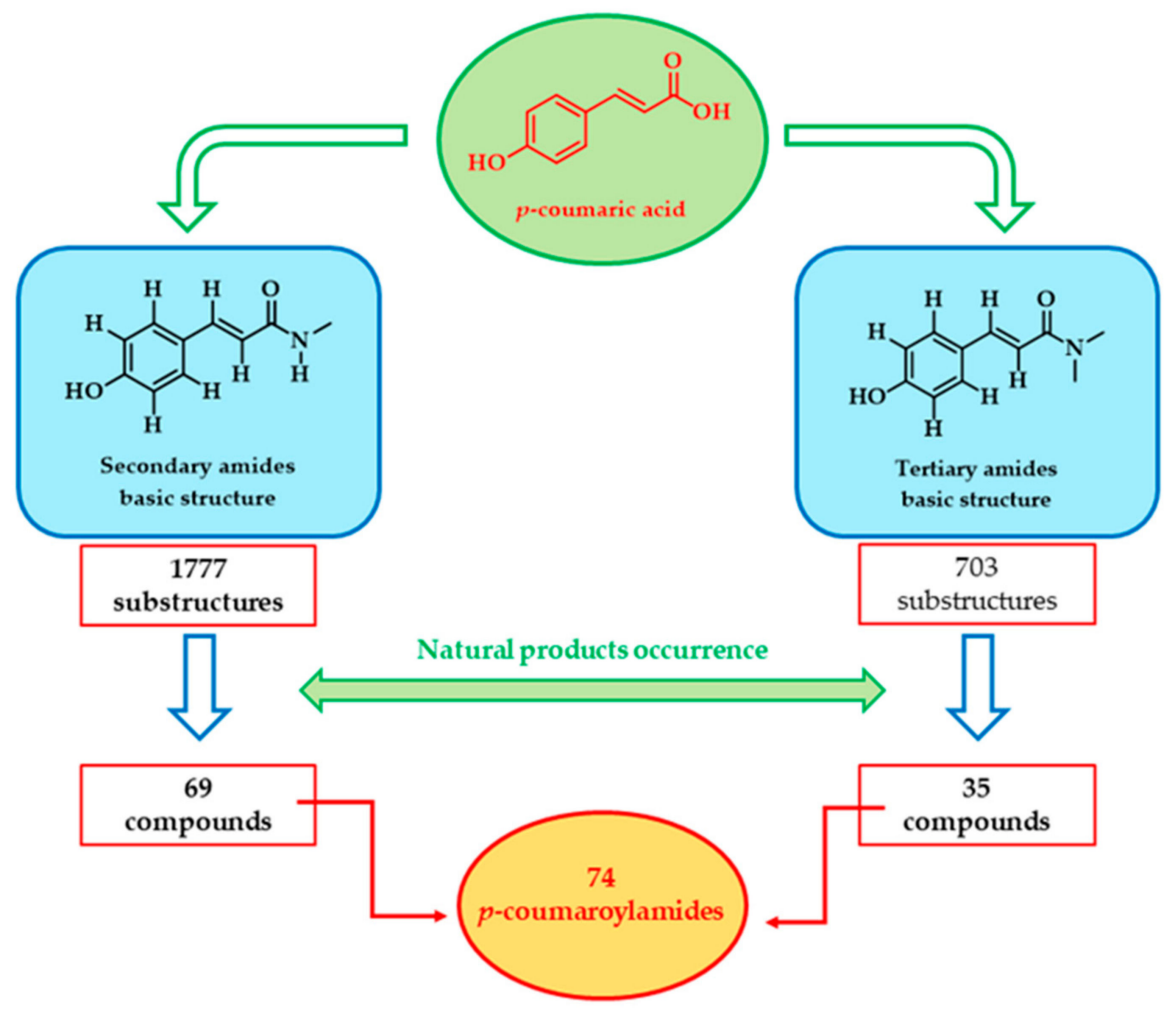
Flowchart of the bibliographic research process.

Scifinder® (Washington, DC, USA) was the main source of bibliographic information. Secondary and tertiary amides of *p*-coumaric acid were identified and retrieved based on the structural formulas depicted in Scheme 1.

A total of 1777 substructures were obtained using the basic structure of secondary amides, and after refinement of the search term using “natural product occurrence” as “reference role”, 69 compounds were identified. Of these compounds, only those extracted from plants, and which are not part of recent reviews were considered. Patents were not considered. Some of the structures belonged to the same compound but had different CAS numbers, depending on whether the stereochemistry was defined or not. The same approach was applied for the tertiary amides, resulting in 35 compounds. The same criteria were applied in the identification of amide derivatives (Scheme 1).

Most of the compounds are present as both *cis* and *trans* isomers. In the text, when no specific configuration is included in the name of the compound, the *trans* isomer is considered.

## 2. *p*-Coumaroyl Agmatine and Its Biosynthesis

**Figure 1:**
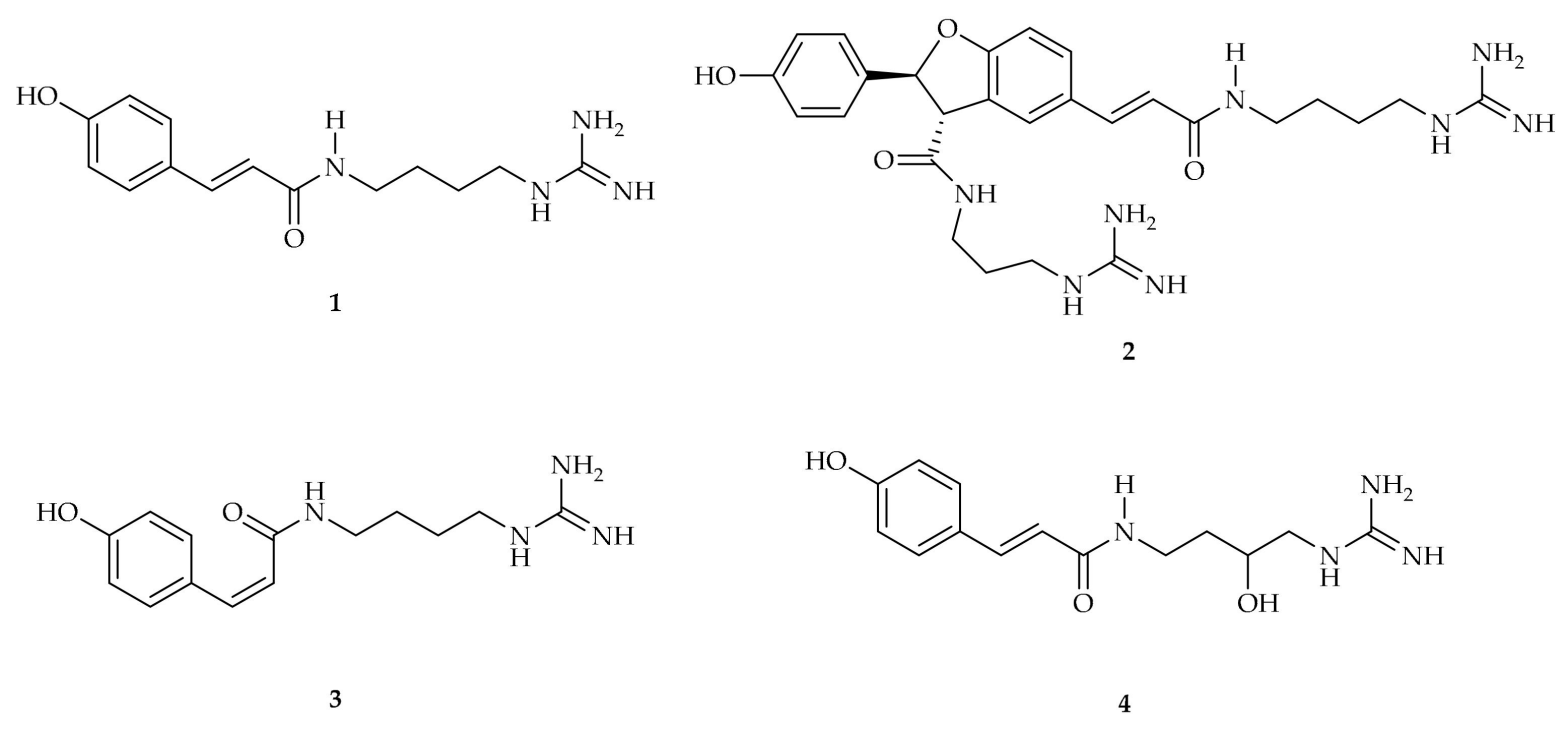
Chemical structures of *p*-coumaroylagmatine **1**, hordatine A **2**, *cis*-*p*-coumaroylagmatine **3,** and *p*-coumaroylhydroxyagmatine **4**.

*p*-Coumaroylagmatine **1** was first isolated in 1965 from young barley shoots (*Hordeum vulgare* L.) by Stoessl [6]. Its biosynthesis was later elucidated by Bird and Smith [7]. An enzyme from extracts of the shoots of barley seedlings has been reported to synthesize coumaroylagmatine from *p*-coumarylCoA and [U-^14^C]agmatine [8]. The dehydrodimer of *p*-coumaroylagmatine, hordatine A **2**, is an antifungal compound that accumulates at high levels in young barley seedlings. It was recently demonstrated that laccase HvLAC enzymes mediate oxidative coupling of *p*-coumaroylagatine **1** during hordatine A **2** biosynthesis [9]. It seems that the ability to biosynthesize hordatines is limited to the genus *Hordeum* (Figure 1).

Compound **1** was identified in rice (*Oryza sativa*) as part of a study aimed at profiling a collection of rice germplasm [10]. It was observed that the overall contents of coumaroyl or feruloylagmatine and putrescine were much higher in indica than in japonica varieties.

Compound **1** was also isolated from *Schrankia leptocarpa* DC. (Mimosaceae), a straggling perennial herb native to tropical South America that was introduced to West Africa [11]. Additionally, *p*-coumaroylagmatine **1** has been identified in several other plants, including *Solanum schimperianum* Hochst (syn. *Solanum careens* Dunal, Solanaceae), *Enterolobium contortisiliquum* (Vell.) *Morong* (Fabaceae), *Brachypodium distachyon* (L.) P. Beauv. (used as a model grass species), *Triticum aestivum* and *Triticum durum* (wheat cultivars), *Ziziphus jujuba* Mill. (Rhamnaceae), *Capsicum annuum* L., *Solanum muricatum* (Solanaceae), *Abrus cantoniensis* Hance and *A. mollis* Hance (Leguminosae), *Camellia sinensis* flowers, and many others. Fungal pathogens affect primary and secondary metabolism in plants; thus, there is a high number of antifungal metabolites, such as *p*-coumaroylagmatine [12].

The *cis* isomer **3** was identified in *Selaginella moellendorffii* Hieron. (Selaginellaceae) [13]. In *Albizzia julibrissin* Durazz, *cis*-*p*-coumaroylagmatine **3** was shown to act as a leaf-opening factor [14] (Figure 1).

*p*-Coumaroylhydroxyagmatine **4** differs from **1** by the presence of a hydroxyl group on the agmatine chain (Figure 1). It has been isolated from barley leaf (*Hordeum vulgare* L.) where it is produced in response to stress conditions such as pathogen infection or climatic conditions [15]. *p*-Coumaroylagmatine **1** and *p*-coumaroyl-3-hydroxyagmatine **4** have been reported to exhibit antifungal activity [16] (Figure 1).

**Scheme 2:**
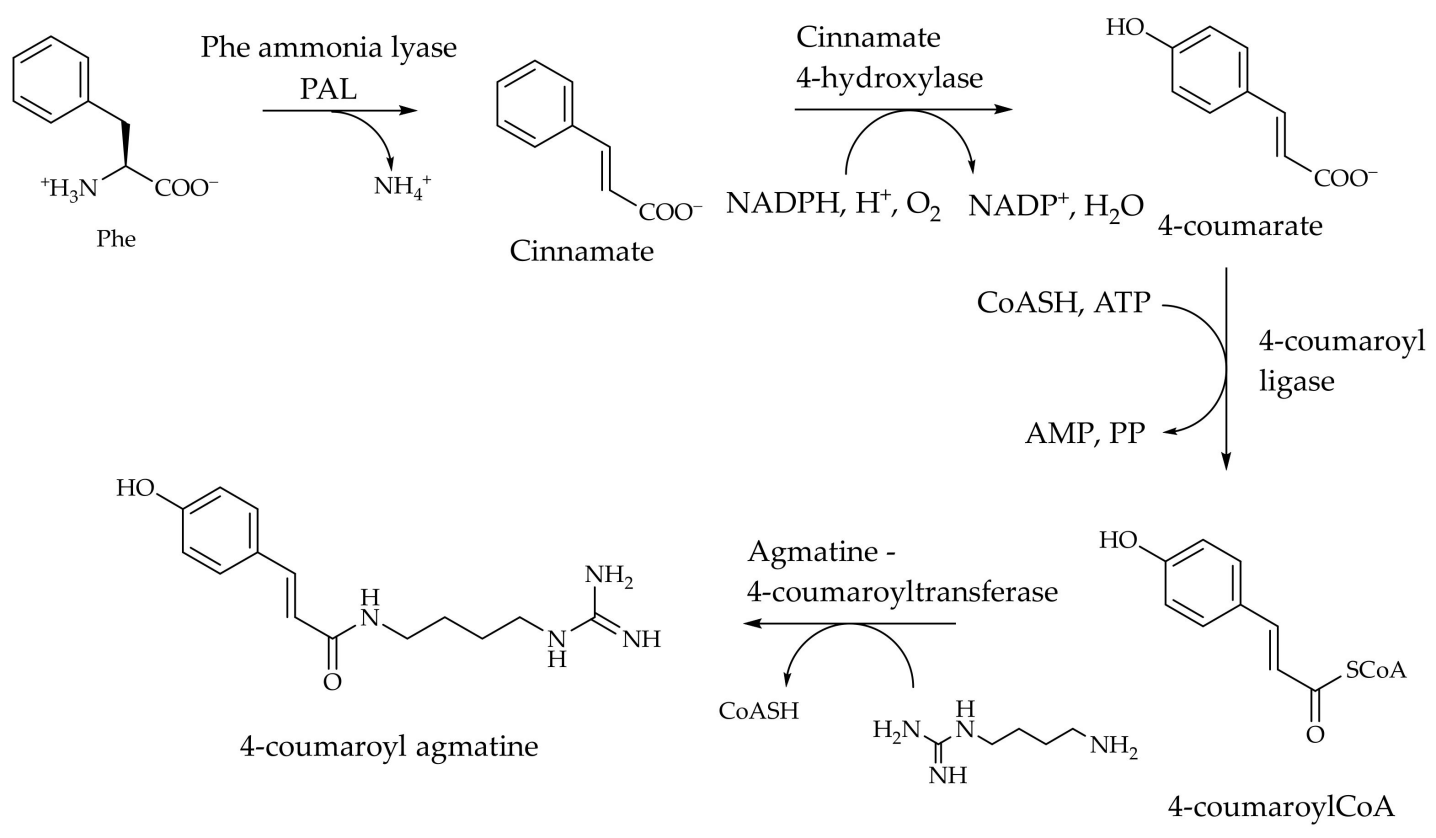
Biosynthesis of *p*-coumaroylagmatine.

The biosynthesis of *p*-coumaroylagmatine has been studied in detail and represents the reference biosynthetic pathway for phenolic acid amides (Scheme 2).

*p*-CoumaroylCoA is a key biosynthetic intermediate involved in the synthesis of many classes of natural products in plants. The key step in its transformation into amides is the final step; namely, acyl transfer from *p*-coumaroylCoA to the amine acceptor. The levels of expression of active *p*-coumaroyl transferases control the amount of amides found in plants, mostly in seeds.

In general, acyl transfer from phenolic acids may be catalyzed by transferases belonging to two different superfamilies: SCPLS (serine carboxypeptidase-like), which exploit β-acetal glucose esters as the activated substrates, as in the case of sinapoyl transfer; and BAHD, whose substrates are acylCoAs of phenolic acids. The BAHD family of acyltransferases was reviewed by D’Auria in 2006 [17] and 2023 [18].

In plants, and particularly in Triticeae such as barley (*Hordeum vulgare* L.) and wheat (*Triticum aestivum* L.), the transfer of coumaroyl groups to agmatine is catalysed by agmatine coumaroyl transferases (ACTs, EC 2.3.1.64). The synthesis of other coumaroyl amides is catalysed by specific enzymes belonging to the same BADH family; however, agmatine coumaroyl transferase is the only member that has been studied in detail and is the only one reported in the BRENDA database [19]. The MetaCyc database also contains records for a serotonin *N*-hydroxycinnamoyl transferase from pepper expressed in transgenic rice [20], and a tyramine *N*-hydroxycinnamoyl transferase from potato [21]. Conversely, UniprotKB [22] contains more records of rice coumaroyl transferases operating on agmatine (entry Q7XPK7), putrescine (entries Q7XXN5 and Q5SMQ0), tryptamine (entries Q8LMI4 and Q338X7), and spermidine (entries Q2QRK9 and Q0ING3).

Two structures of coumaroyl transferases are available in the Protein Data Bank, and both have been resolved recently by Masayuki Sue et al. The first structure (PDB id. 7CYS) is from barley [23] and the other (PDB id. 7DTP) is from wheat [24]. The structures were obtained by the authors during a study aimed at elucidating the mechanisms of coumaroyl transfer in multiple species of barley, wheat, and rice. The accumulation of coumaroylagmatine and other coumaroyl amides and their structural diversities represent an important defense system available to plants against microorganisms and insects. Therefore, knowledge of the mechanisms controlling the biosynthesis of such compounds may be helpful in the development of sustainable agricultural practices. The studies included measuring the concentration of coumaroylagmatine in seeds; isolating putative genes encoding transferases identified by a BLAST (as implemented in UniProt) search; measuring transcription levels, expression of the enzymes, and analysis of their activity; and finally crystallization. Two enzymes are encoded in the genomes of barley and wheat (ACT1 and ACT2) as a result of a probable gene duplication; however, ACT1 is transcribed at high levels in both barley and wheat. Other transferase genes are present but not expressed in intact plants, while they are reported to be overexpressed in plants infected by fungi. Seven enzymes from different species were studied with four acylCoAs (*p*-coumaroyl, feruloyl, caffeoyl, and cinnamoyl) and agmatine as the acceptor. The enzymes from rice were less proficient, while the most active one, which was also the most expressed, was Hv-ACT1-1. It is active on all the acyl-CoAs, and the best substrate at saturation is cinnamoylCoA, with k_cat_ as high as 110 s^−1^, about twice that of coumaroylCoA. However, under substrate-limiting conditions, its performance is almost identical on all substrates, except feruloylCoA. The k_cat_/K_M_ for coumaroylCoA is 55.4 L·s^−1^·μmol^−1^.

As for the acceptor amines, these enzymes were tested against agmatine, putrescine, spermine, and spermidine, and all were very selective toward agmatine, with negligible activities against spermine and spermidine.

**Figure 2:**
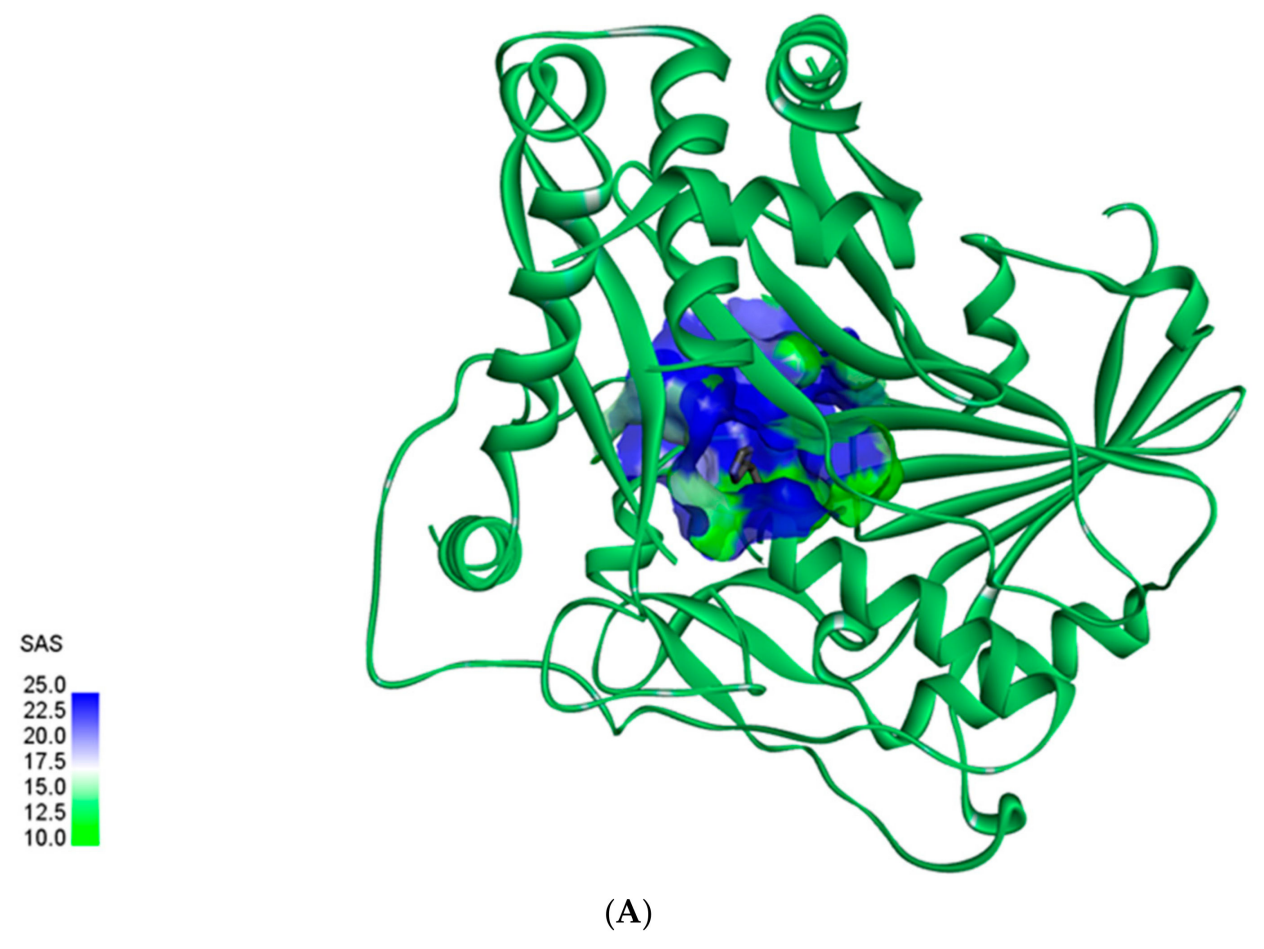

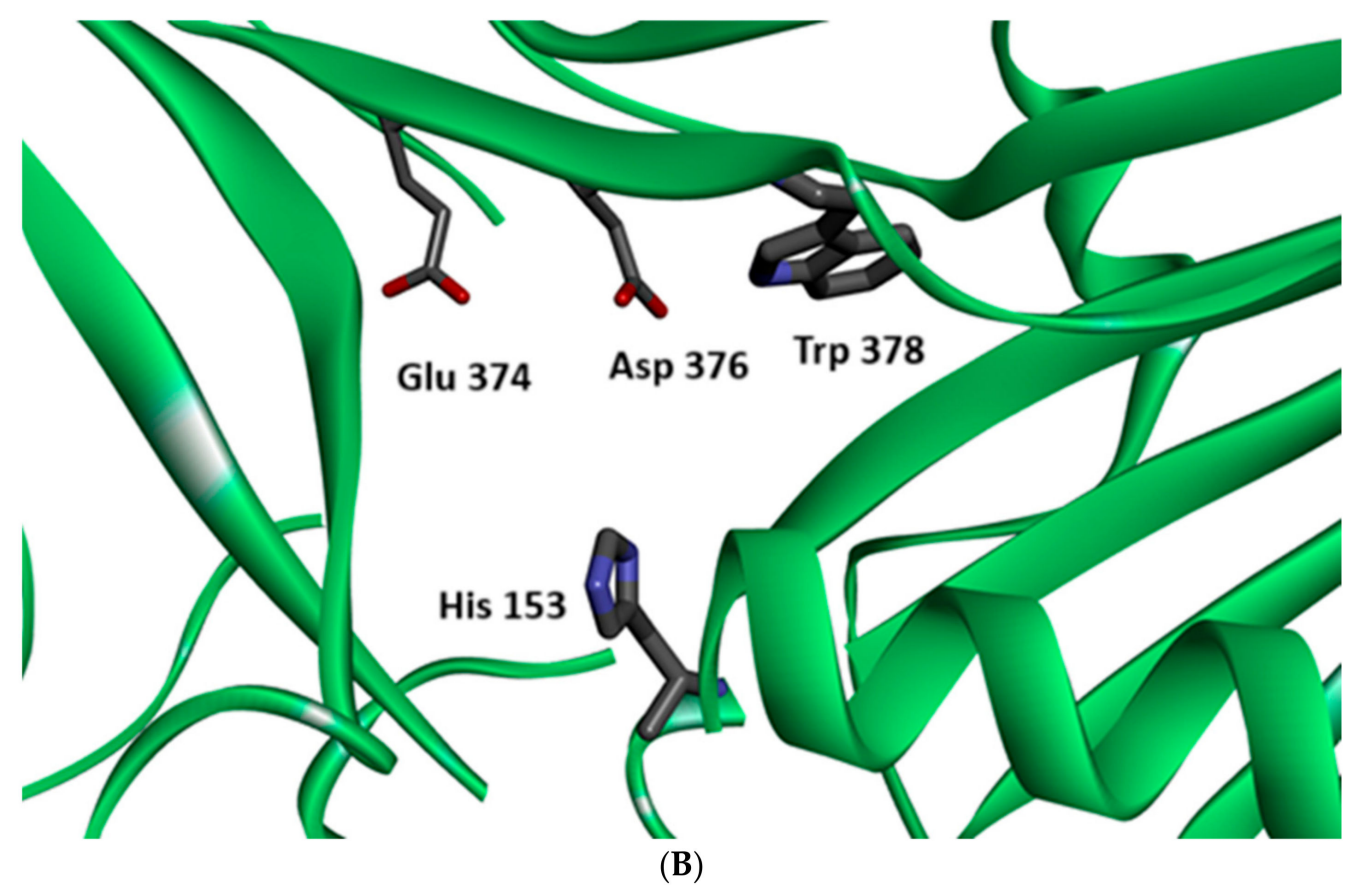
(**A**) View of the entry channel to the catalytic site of ACT2; its solvent-accessible surface (SAS) is reported, colored according to the % accessibility of its amino acid residues. His 153 can be seen at the bottom of the site. (**B**) Detailed view of the catalytic site, with the residues involved in catalytic activity highlighted.

Agmatine-4-coumaroyl transferase is the only coumaroylCoA transferase that has been studied in detail. The mechanism and structures of the enzymes involved in the conversion of *p*-coumaroylCoA into other amines to generate the amides reported in this review are not known; however, the general picture is likely similar. In addition, ACT1 and ACT2 have a highly conserved catalytic site structure: a histidine (153 in ACT2) plays the key role, possibly via nucleophilic or mechanistic base catalysis. The histidine is located at the bottom of the catalytic site (Figure 2A). Aspartate 374 and glutamate 376 are also essential for substrate recognition (probably by complementing the charge of agmatine, and/or keeping the nucleophilic nitrogen neutral), while tryptophan 378 may allow interactions with the aromatic ring of *p*-coumaroylCoA (Figure 2B).

## 3. Secondary Amides

### 3.1. Isolation, Characterization, and Role in Plants

**Figure 3:**
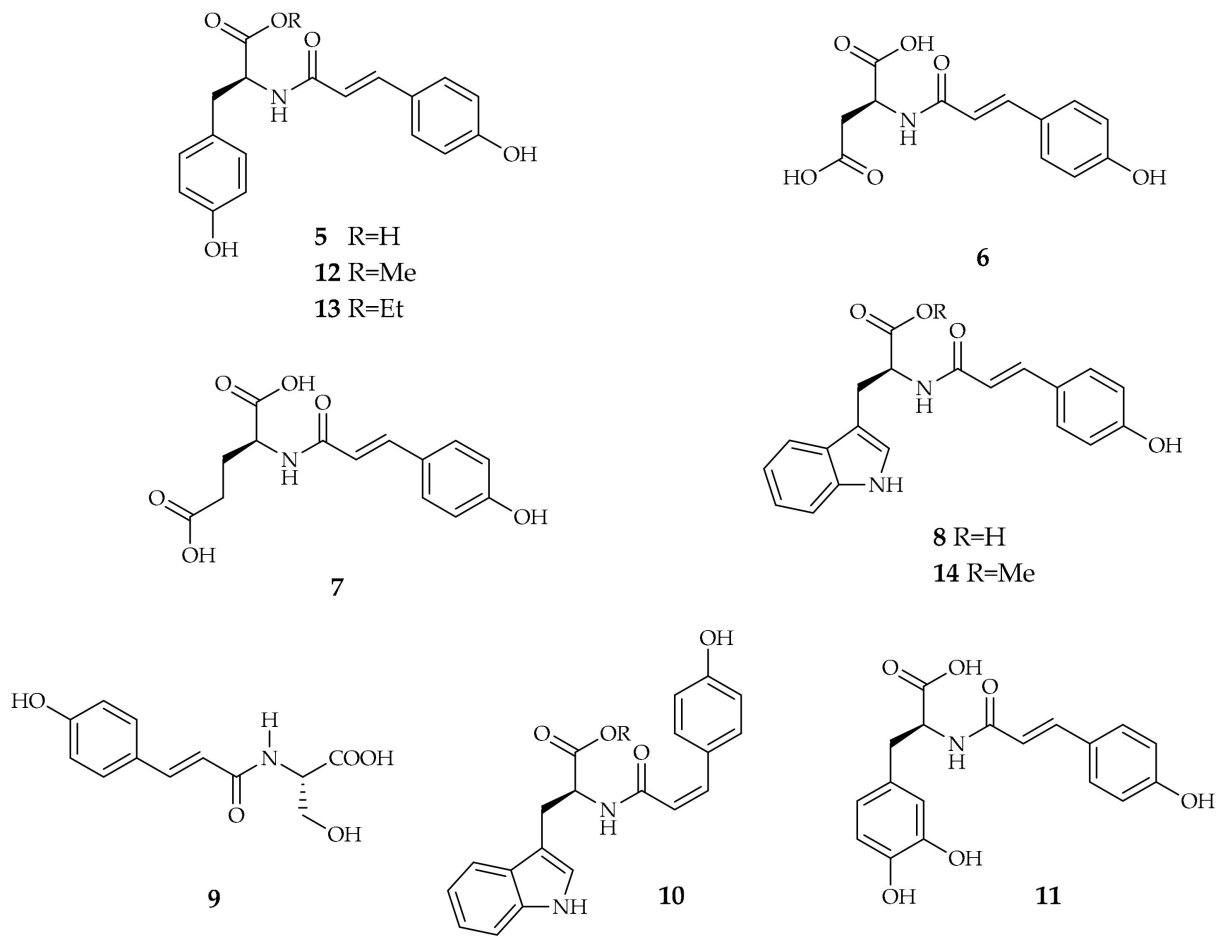
Chemical structures of *p*-coumaroyltyrosine **5**, *p*-coumaroylaspartate **6**, *p*-coumaroylglutamate **7**, *p*-coumaroyltryptophan **8**, *p*-coumaroylserine **9**, *cis-p*-coumaroyltryptophan **10**, *p*-coumaroyl-DOPA **11**, *p*-coumaroyltyrosine methyl ester **12**, *p*-coumaroyltyrosine ethyl ester **13**, and *p*-coumaroyltryptophan methyl ester **14**.

*p*-Coumaric acid is often found in nature conjugated with several other compounds to form amides. A first class of secondary amides containing the *p*-coumaroyl moiety is obtained from conjugation of *p*-coumaric acid with amino acids. The *p*-coumaroylamino acid derivatives are predominantly found in *Theobroma cacao* and *Coffea canephora*, although they are also present in other plants. The most naturally occurring *p*-coumaroylamino acids discussed in this section are *p*-coumaroyltyrosine **5**, *p*-coumaroylaspartate **6**, *p*-coumaroylglutamate **7**, *p*-coumaroyltryptophan **8**, and *p*-coumaroylserine **9**. The *cis-p*-coumaroyltryptophan **10** has been identified in *Amaranthus blitum* L., while the non-proteinogenic *p*-coumaroyl-DOPA **11** has been found in cocoa nibs as well as in cocoa powder [25] (Figure 3).

**Figure 4:**
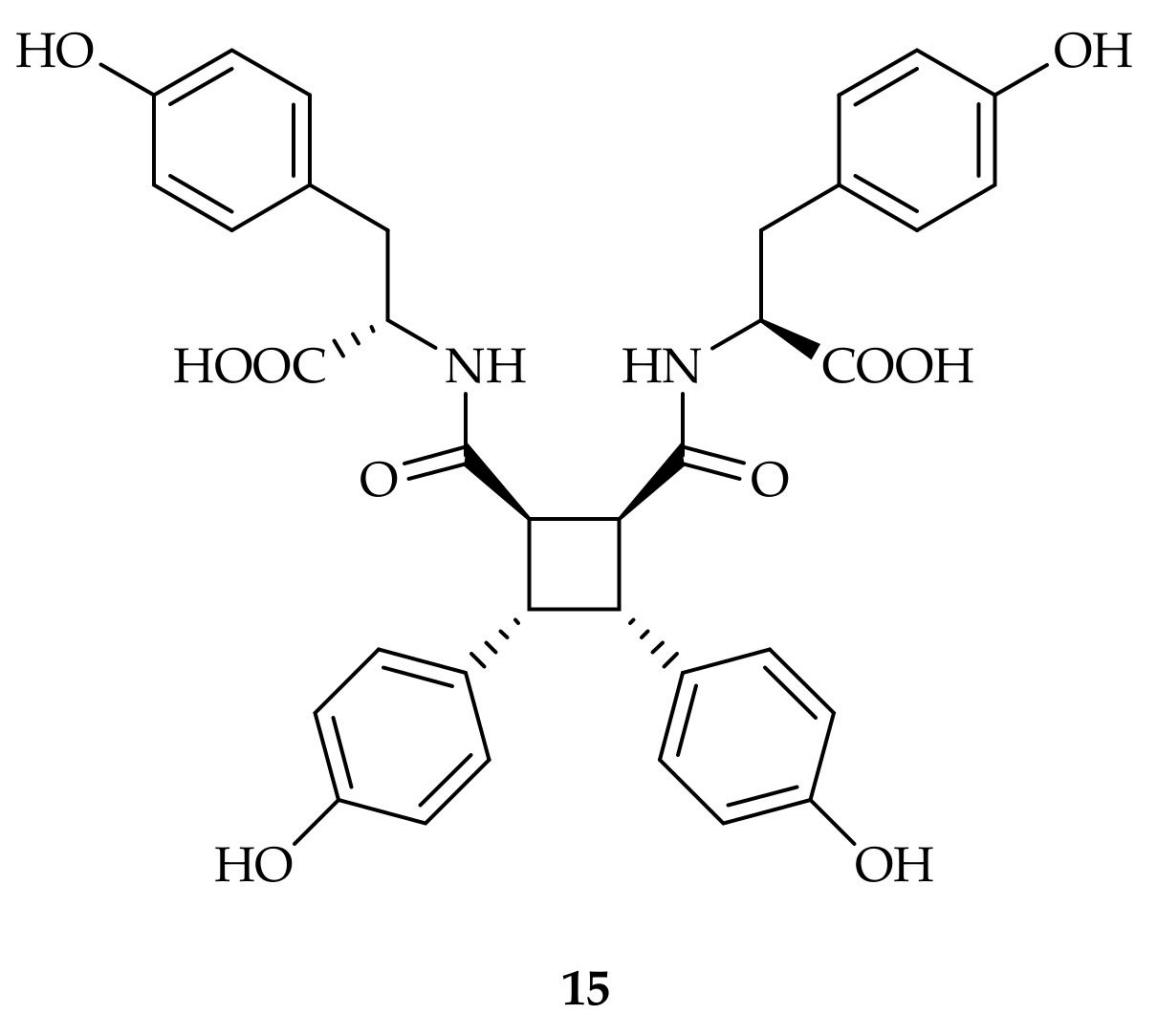
Chemical structure of abrusamide A **15**.

*p*-Coumaroyltyrosine **5** is the major compound in the leaves of Abri herba, identified in amounts ranging from 0.75 to 6.36 mg·g^−1^, depending on the origin of the samples. It is also found in *Abrus mollis* and *Abrus cantoniensis*, whose leaves are rich in amides [26]. *Abrus mollis* leaves are also rich in other compounds, with truxillate and truxinate skeletons having a characteristic cyclobutane ring derived from the [2 + 2] dimerization of both *cis-* and *trans-p*-coumaroyltyrosine **5**. Figure 4 shows the structure of one of these compounds, named Abrusamide A **15**, whose configuration was recently reassigned [27]. One of the isomers of Abrusamide A **15** has also been identified in *Coffea canephora* by LC-MS, while it seems to not be present in *Coffea arabica* [4].

**Figure 5:**
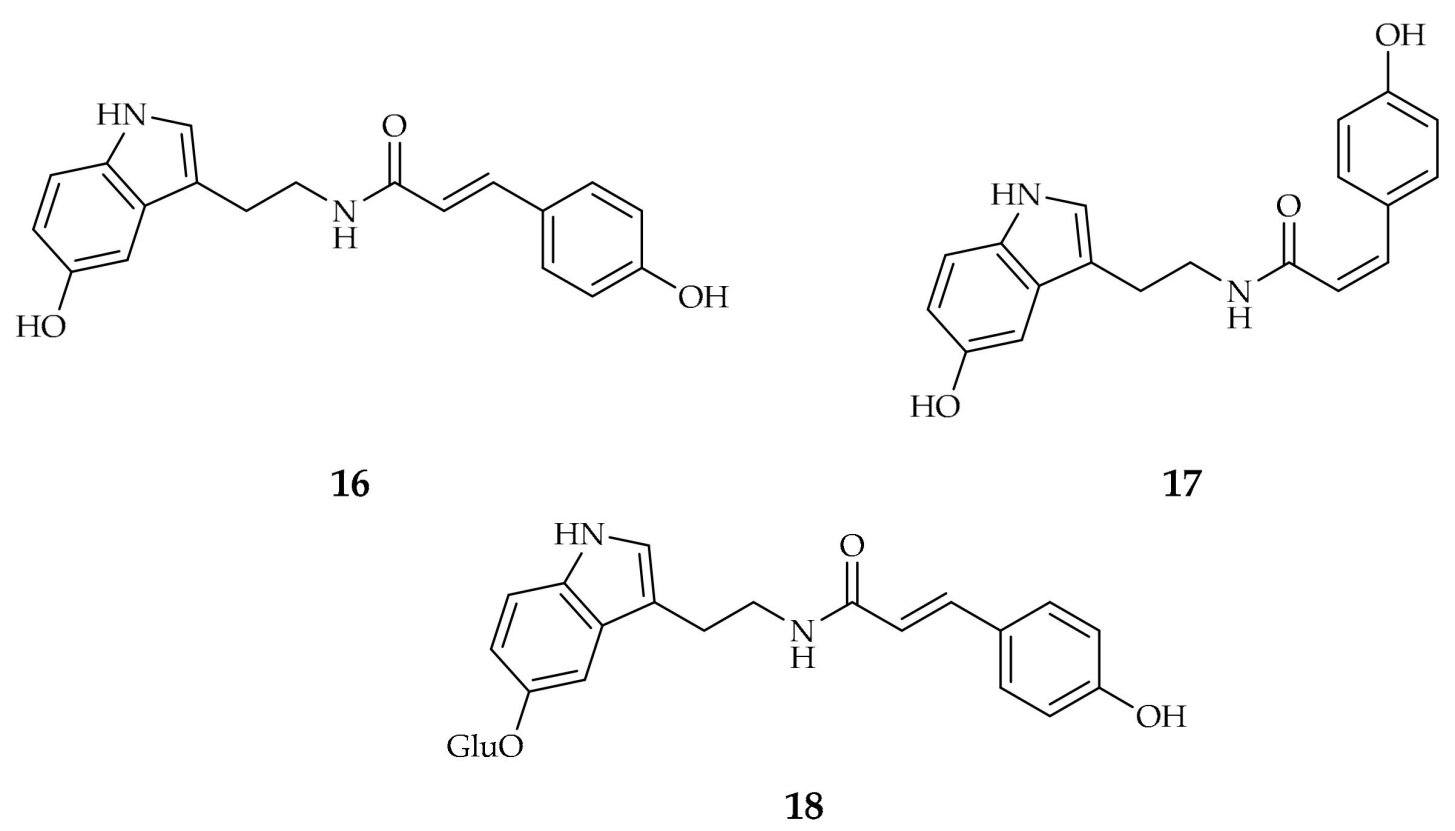
Chemical structures of *p*-coumaroylserotonin **16**, *cis-p-*coumaroylserotonin **17**, and *p*-coumaroylserotonin-7-glucoside **18**.

*p*-Coumaric acid can also be conjugated with serotonin; the resulting derivative is widely present in different plant species as both *p*-coumaroylserotonin **16** and *cis-p*-coumaroylserotonin **17** (Figure 5). Compound **16** has been isolated from Japanese Barnyard millet (*Echinochloa utilis*) grains [28], Proso millet (*Panicum miliaceum* L.), a cereal crop of Gramineae, one of the most important and ancient domesticated crops in the world [29], safflower (*Carthamus tinctorius* L.) seeds [30], *Centaurea nigra* (Asteraceae), *Centaurea vlachorum* Hartv [31], *Croton echioides* [32], *Croton meyharthii* [33], *Zea mays* [34], *Phragmites australis* (Cav.) Trin. ex Steud [35], *Sasa quelpaertensis* Nakai (Poaceae), *Arundo donax* L. [36], *Ptycopetalum olacoides* [37], and *Anthocleista vogelii* Planch. Usually, extraction is performed with a hydroalcoholic mixture and both methanol and ethanol can be used. Subsequent fractionation of the extract leads to the isolation of compounds **16** and **17**.

The corresponding *cis* isomer **17** has been identified in *Centaurea montana* (Asteraceae) [38], Konnyaku (*Amorphophallus konjac*) [39], and *Homalomena occulta* (Lour.) Schott (Araceae) [40].

The glucoside derivative of *p*-coumaroylserotonin **16**, compound **18**, has been isolated from *Carthamus tinctorius* L. and *Carthamus oxyacantha* M. Bieb. [41] (Figure 5). In this case, the extraction was performed on the defatted seeds with 80% (*v*/*v*) aqueous methanol solution containing 0.1% formic acid using a vortex mixer and an ultrasonic bath, and the identification was made by means of LC–HRMS/MS^2^.

**Figure 6:**
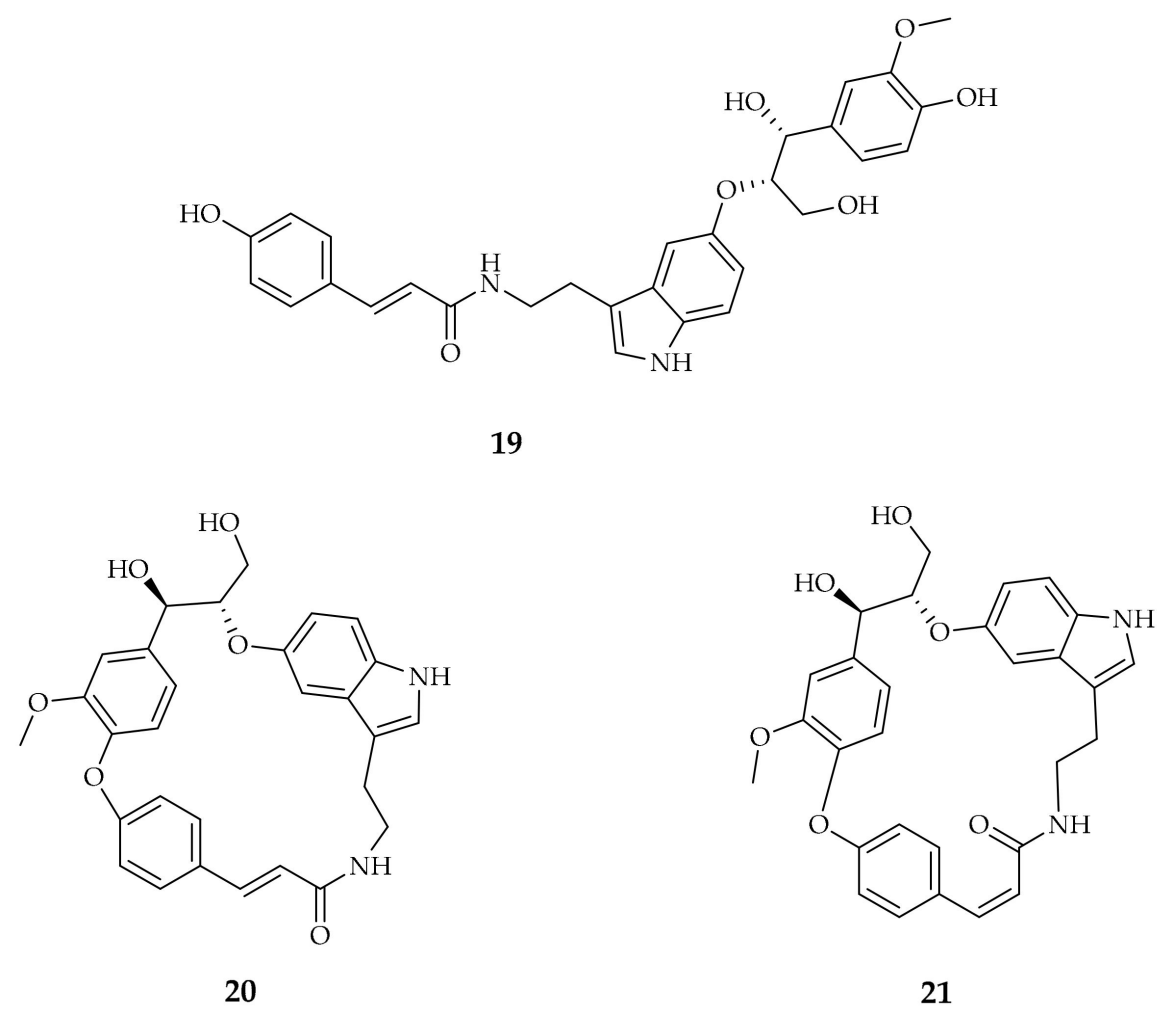
Chemical structures of ipobscurine B **19**, ipobscurine C **20**, and ipobscurine D **21**.

Other serotonin derivatives with *p*-coumaric acid include ipobscurine B **19**, which is a *p-*coumaroyl amide of a serotonin ether with a tri-hydroxypropane containing a further phenolic system. It is found in *Ipomea obscura* L. Ker-Gawl., a very common and widespread perennial herb. Oxidative coupling between the two phenolic systems of the molecule occurs in the plant, leading to two macrocyclic compounds, named ipobscurine C **20** and D **21 [42]** (Figure 6). Extraction was performed on the defatted seeds using MeOH at room temperature and the crude extract obtained was subsequently extracted with EtOAc from a 2% tartaric acid aqueous solution. Several fractionations using both column chromatography and HPLC afforded the desired compounds, which were characterized by means of NMR spectroscopy.

**Scheme 3:**
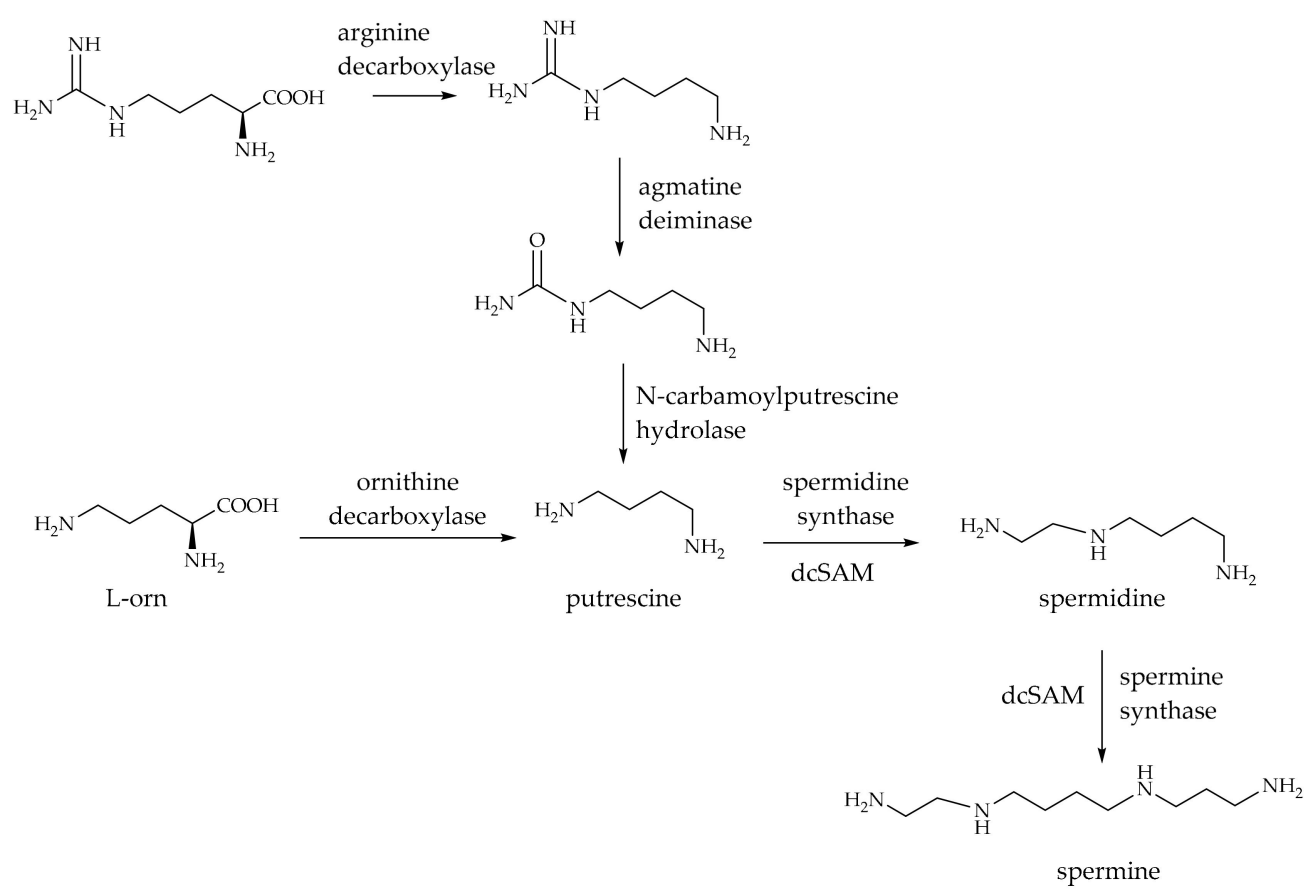
Biosynthetic pathway of polyamines in plants.

A further class of *p*-coumaroyl amides consists of the conjugation of *p*-coumaric acid with different polyamines, which are usually synthesized in plants either from L-ornithine and L-methionine or from arginine, as indicated in Scheme 3. The arginine pathway also involves decarboxylation, but requires additional hydrolysis reactions to cleave the guanidine portion. The aminopropyl groups are then transferred from a decarboxylated SAM (dcSAM) to putrescine in the subsequent steps. These reactions first give spermidine and then spermine.

The polyamines so formed can be conjugated with different hydroxycinnamic acids, forming one of the largest classes of plant-specialized secondary metabolites, the phenolamides (PAs), which play important roles in plant growth, reproduction, and biotic and abiotic stress resistance. The polyamines can be mono- or polysubstituted with *p*-coumaric acid, giving origin to several derivatives. These compounds are present in foxtail millet, rice, wheat, and maize, and their related food products, as well as in other nonfood-related plants. Additionally, other species, including *Coix lacryma-jobi*, *Orostachys japonicus* [43], *Artemisia caruifolia* [44], *Carthamus tinctorius* [45], *Scopolia tangutica* [46], and *Thyrocarpus glochidiatus* [47] are known to contain high levels of polyamine conjugates and are employed as medicinal plants due to their properties.

Moreover, polyamines and their conjugates with hydroxycinnamic acids are mostly found in bee pollen, which contains digestive enzymes secreted by bees and undergoes a fermentation process in order to enhance its stability and digestibility. Due to this transformation induced by bee enzymes, the hydroxycinnamoyl polyamines in bee pollen will not be reported since the present review is focused only on compounds present in plants.

**Figure 7:**
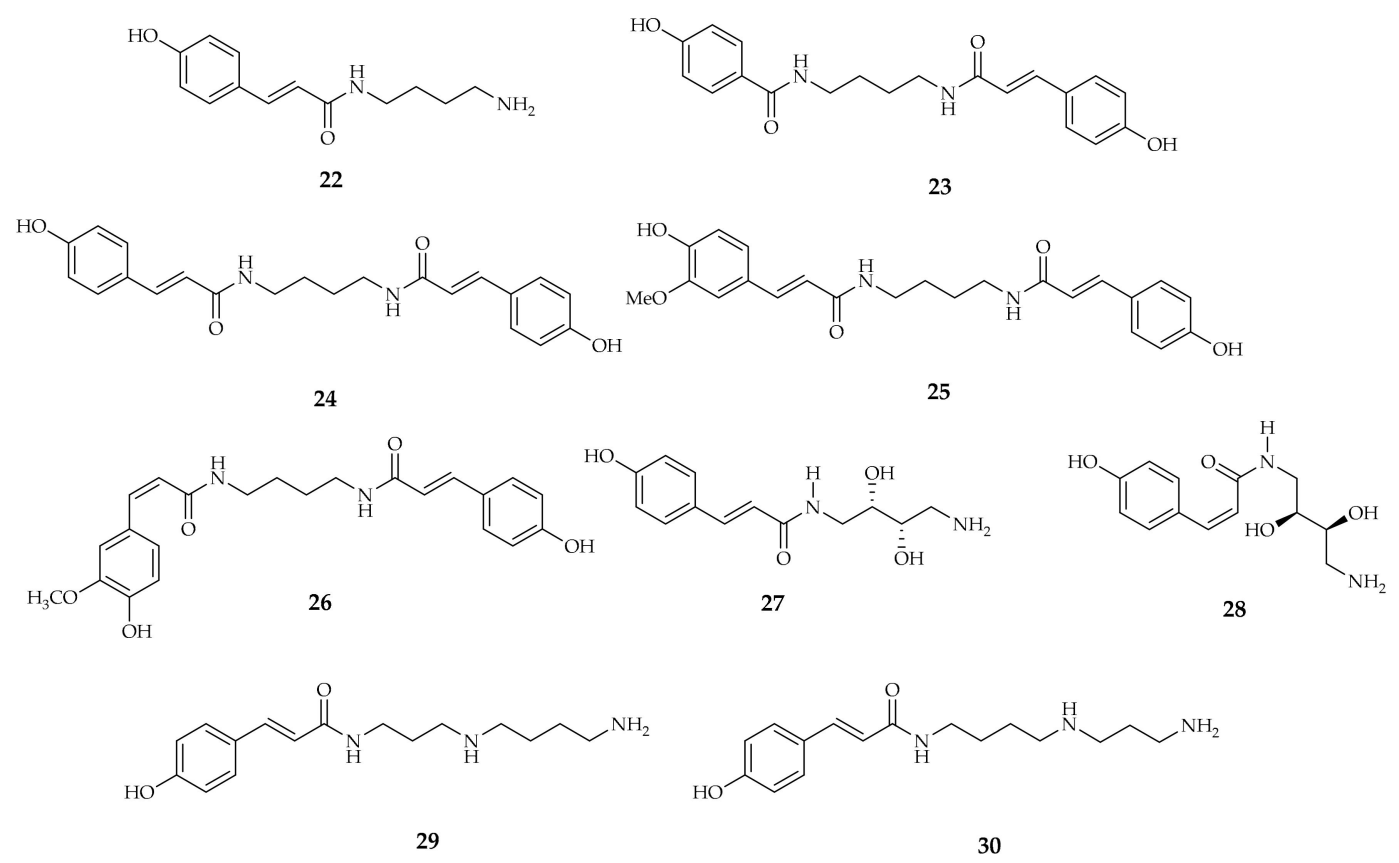
Chemical structures of coumaroylputrescine **22**, glochidiatusamide **23**, di-*p*-coumaroylputrescine **24**, feruloyl-*p*-coumaroylputrescine **25**, (*Z*)-feruloyl-(*E*)-*p*-coumaroylputrescine **26**, pharnilatin A **27**, pharnilatin B **28**, *N*^1^-coumaroylspermidine **29**, and *N*^10^-coumaroylspermidine **30**.

Various mono- and di-substituted putrescine-derived hydroxycinnamic acid amides (HCAAs) were found in tea flowers (*Camelia sinensis*) and the *p*-coumaroylputrescine derivatives **22**–**24** have been identified by UHPLC/TOF-MS [1] (Figure 7).

Compound **22** is one of the antioxidant metabolites produced in response to potato tuber wounding recognized in the most potent antioxidant fractions using the ABTS radical scavenging assay and LC-MS with TOF-MS for compositional analysis [48].

Compound **22** is also one of the compounds that accumulates in response to attack by chewing insects. In 2016, a higher mortality was observed in the sucking insect brown planthopper (*Nilaparvata lugens*) raised on 15% sugar solution containing *p*-coumaroylputrescine **22** or feruloylputrescine, at concentrations similar to those elicited by heavy BPH attack in rice, in comparison to those raised on sugar diet alone [49]. Another putrescine derivative is compound **23**, which is also called glochidiatusamide because it was isolated from *Thyrocarpus glochidiatus*, a plant present only in China and used for medical purposes [47].

Fiber-rich by-products, including cereal bran, fruit peels, and vegetable residues, are a source of *p*-coumaroyl polyamine derivatives. HPLC and MS are mostly used to identify and quantify these compounds. Corn bran, which is an important byproduct of the corn dry-milling industry, is rich in several functional lipid constituents, including polyamineconjugates such as diferuloylputrescine, dicoumaroylputrescine **24**, and *p-*coumaroylferuloylputrescine **25** (Figure 7).

Compound **25** is involved in plant defense against the leaf folder, *Cnaphalocris medinalis*, a major pest in rice cultivation. By increasing cell wall strength, modulating oxidative stress, and modulating the signaling cascades controlled by jasmonate, compound **25** protects plants from pest attacks [50].

Compound **24** also contributes to plant growth because of its ability to induce NO release. NO is an antioxidant, plant-growth modulator, and antiapoptotic regulator; however, its enzymatic production in plants can be slow, thus a non-enzymatic mechanism is required to accumulate NO. The capacity of compound **24** to induce NO is lower than those of caffeoylputrescine and feruloylputrescine. This is probably correlated to the different antioxidant abilities of caffeoyl and feruloyl amides, which are generally higher than those of *p*-coumaroyl amides [51].

The *cis* isomer **26** has been isolated from the aerial parts of *Nanophyton erinaceum* (Pall.) Bunge (Chenopodiaceae), widely distributed in central and western Asia [52]. Its structure was elucidated using a combination of spectroscopic methods (ESI-MS, ^1^H and ^13^C NMR) and comparison with data in the literature. The configuration *cis* was assigned by ^1^H NMR data comparing coupling constants with the ones of compound **25** (Figure 7).

Two new phenolic amides, pharnilatins A **27** and B **28**, were isolated using 50% EtOH extraction from the seeds of *Pharbitis nil* L. (Convolvulaceae), traditionally used as a purgative drug in Korea, China, and Japan [53]. These new compounds possess a *p*-coumaroyl unit with a structurally unique side chain—(2*S*,3*S*)-2,3-dihydroxyputrescine. The chemical structures and absolute stereochemistry of the new compounds were determined using spectroscopic analyses, including 1D and 2D NMR experiments and chemical reactions (Figure 7).

*N*^1^-coumaroylspermidine **29** and *N*^10^-coumaroylspermidine **30** are spermidine derivatives found in *Solanum dulcamara* L. [54]. Their ability to induce NO production is lower than those of their caffeoyl and feruloyl counterparts, probably due to the different antioxidant power of the *p-*coumaroyl amides compared to the caffeoyl or feruloyl amides [51] (Figure 7).

**Figure 8:**
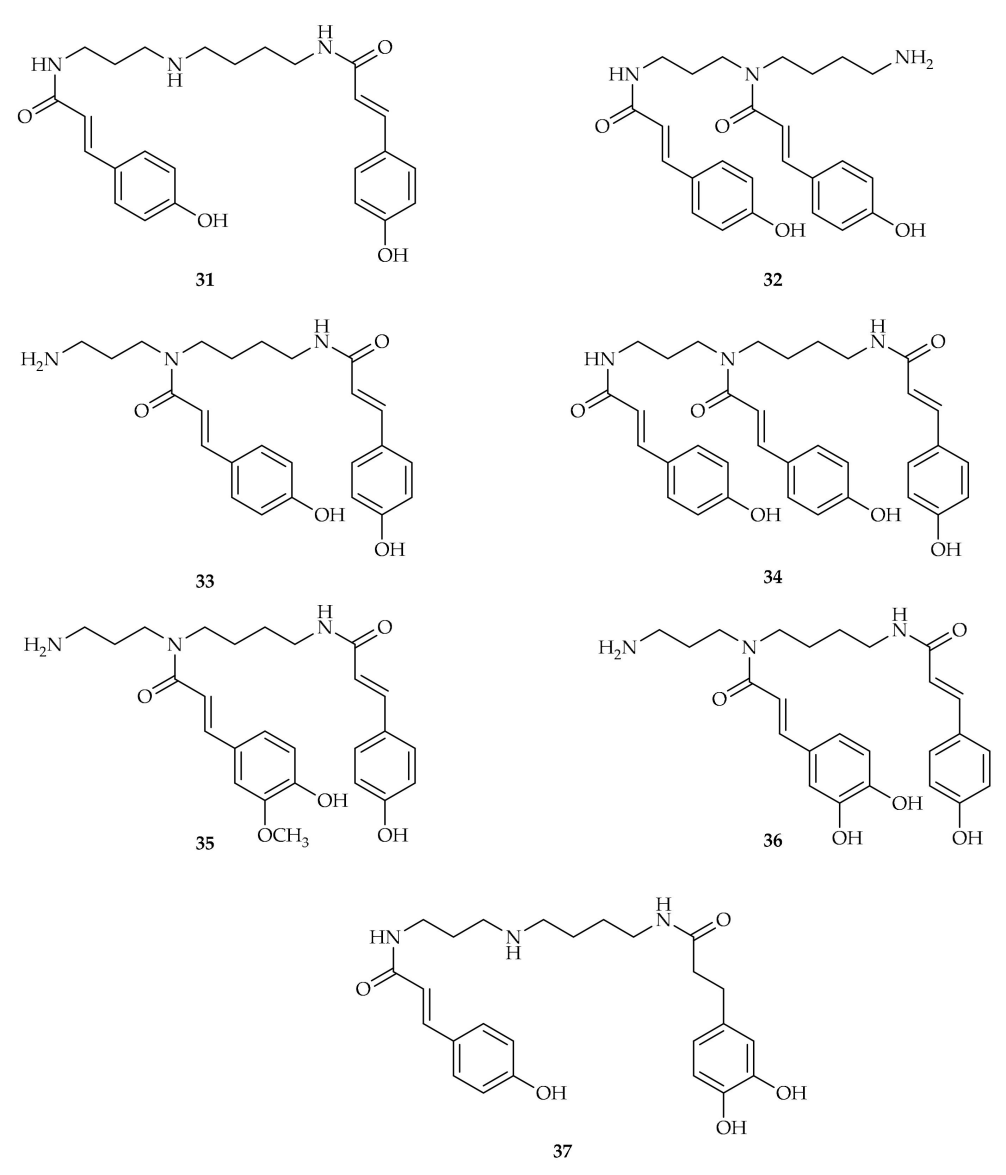
Chemical structures of *N*^1^,*N*^10^-di-*p*-coumaroylspermidine **31**, *N*^1^,*N*^5^-di-*p*-coumaroylspermidine **32**, *N*^5^,*N*^10^-di-*p*-coumaroylspermidine **33**, *N*^1^,*N*^5^,*N*^10^-tri-*p*-coumaroylspermidine **34**, *N*^5^-*p*-coumaroyl-*N*^10^-feruloylspermidine **35**, *N*^5^-*p*-coumaroyl-*N*^10^-caffeoylspermidine **36**, and *N*^1^-*p*-coumaroyl -*N*^10^-dihydrocaffeoylspermidine **37**.

Similar to most of the cinnamoyl amides already described, *N*^1^,*N*^10^-di-*p*-coumaroylspermidine **31** has a defensive role in plants (Figure 8). In fact, compound **31** levels increase in the leaves of leaf folder-infested plants, similar to what is observed for compound **25**, suggesting its defensive role against herbivorous insects [50]. Compound **31** acts by cross-linking cell wall components, making tissues resistant to insect-feeding, scavenging free reactive oxygen species, and regulating the jasmonate-mediated resistance pathway [50]. Moreover, compound **31** is the penultimate precursor of lunarine, an alkaloid present only in the seeds of *Lunaria annua*, as demonstrated using a synthetic labelled di-*p*-coumaroylspermidine [55].

*N*^1^,*N*^5^-dicoumaroylspermidine **32** has been identified as one of the endogenous bitter compounds in extruded corn puffed products that contribute to bitterness perception at the concentration reported in the saliva after mastication of the extruded products [56] (Figure 8).

Compound **32** is also present in the pollen of several plants, including *Pterocarya fraxinifolia* (Lam.) Spach [57] and genera *Alnus*, *Betula*, *Corylus*, and *Quercus* [58].

In 1995, Hesse et al. isolated compound **32**, *N*^5^,*N*^10^-di-*p*-coumaroylspermidine **33**, and *N*^1^,*N*^5^,*N*^10^-tri-*p*-coumaroylspermidine **34** from anthers of *Aphelandra tetraffona* and *A. chamissoniana* (Acanthaceae) [59]. Compounds **33**, **35**, and **36** were also extracted from foxtail millet bran, a by-product of the milling process, using deep eutectic solvents [60] (Figure 8).

To improve the analysis of HCAAs, including putrescine and spermidine conjugates, in 2008, Li et al. proposed a deep annotation method using UHPLC-HRMS using an in silico database of HCAAs. The in silico database was constructed by inserting predicted structures, fragmentation patterns, and retention times of many different HCAAs, with the possibility of detecting both known and unknown hydroxycinnamic acid derivatives without knowledge of their distribution [61]. Quantitative structure-retention relationship (QSRR) models are used to predict the retention times of mono-, di- and tri-HCAAs with high accuracy. MS/MS fragmentation patterns of HCAA derivatives were developed in detail and retention time prediction combined with fragmentation data improved the identification of these secondary metabolites. The method was applied to the identification of HCAAs in maize, wheat and rice seeds, rice roots, and rice and tobacco leaves. Using this method, high levels of compounds **31** and **37** were detected in maize seeds [61]. Several alkaloids were identified in *Scopolia tagutica* roots, including the spermidine derivative *N*^1^-*p*-coumaroyl -*N*^10^-dihydrocaffeoylspermidine **37** [46] (Figure 8).

**Figure 9:**
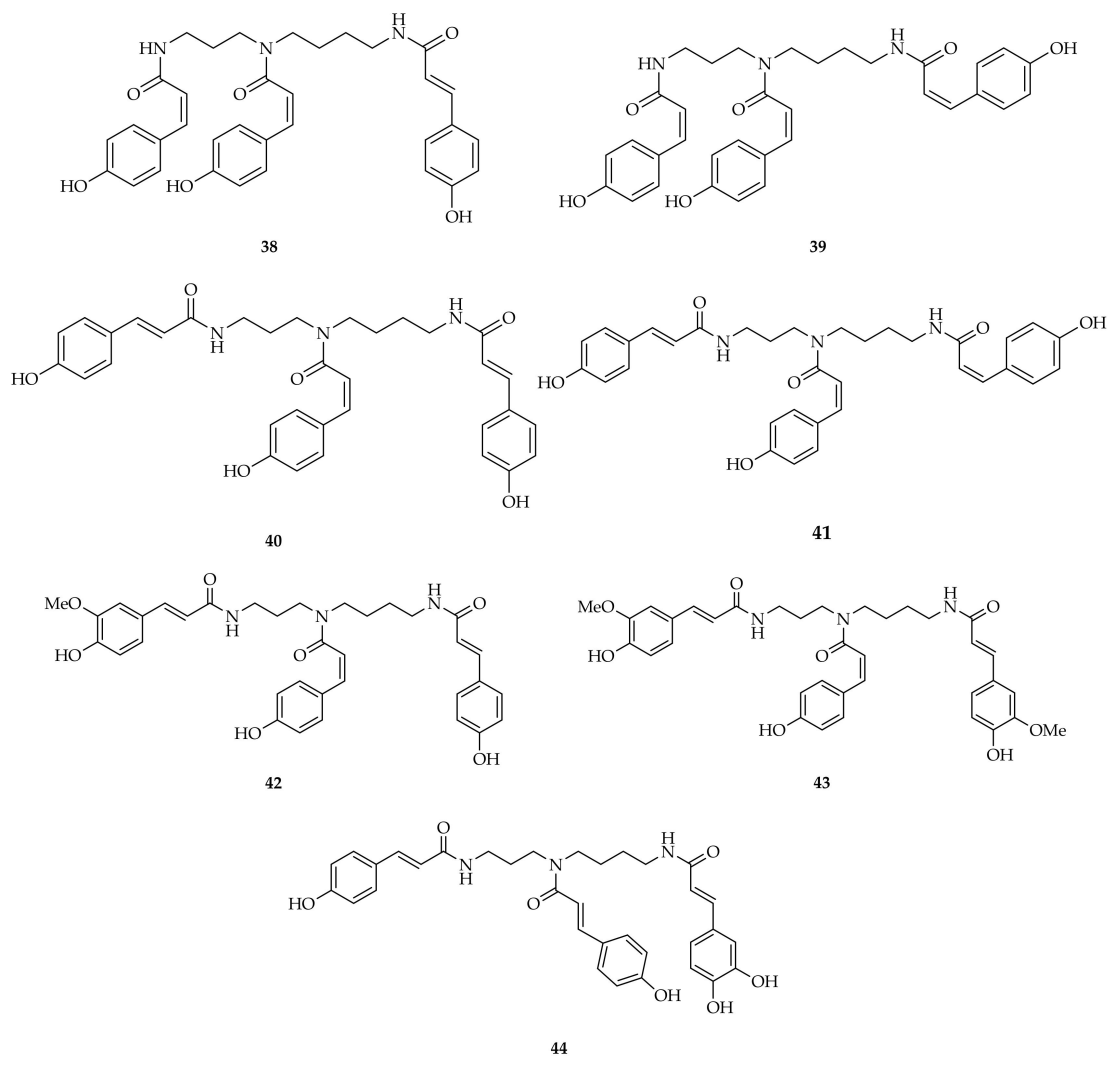
Chemical structures of (*Z*)-*N*^1^,*N*^5^-(*E*)-*N*^10^-tri-*p*-coumaroylspermidine **38**, (*Z*)-*N*^1^,*N*^5^,*N*^10^-tri-*p*-coumaroylspermidine **39**, (*E*)-*N*^1^-(*Z*)-*N*^5^-(*E*)-*N*^10^-tri-*p*-coumaroylspermidine **40** (safflospermidine A)**,** (*E*)-*N*^1^-(*Z*)-*N*^5^- (*Z*)- *N*^10^-tri-*p*-coumaroylspermidine **41** (safflospermidine B), *N*^5^,*N*^10^-di-*p*-coumaroyl-*N*^1^-feruloylspermidine **42** (keayanidine A), *N*^5^-*p*-coumaroyl-*N*^1^,*N*^10^-diferuloylspermidine **43** (keayanidine B), and *N*^1^,*N*^5^-di-*p*-coumaroyl-*N*^10^-caffeoylspermidine **44**.

Compound **34** and three other *p*-coumaroylspermidines (**38**–**40**) were identified in dried safflower residues in amounts of 64.86 ± 0.41% using HPLC [62]. Extraction was performed with 75% EtOH and subsequent fractionation was conducted using RP-C18 column chromatography (Figure 9).

Compound **38** was isolated from *Carthamus tinctorius* L. (Compositae), geologically distributed in all regions of China [63] (Figure 9). A complicated procedure consisting of different extractions and fractionations was necessary to isolate compound **38** as a pure compound. Elucidation of its structure was conducted using IR and NMR analysis.

Compound **39**, safflospermidine A **40**, compound **34**, and safflospermidine B **41** were also isolated from the florets of *Carthamus tinctorius* L. [45] (Figure 9). To identify and correctly assign their structure, bidimensional NMR, especially HMBC, was used.

Different hydroxycinnamic acids are present in spermidine derivatives found in the methanolic root extract of *Microdesmis keayana*. The compounds *N*^5^,*N*^10^-di-*p*-coumaroyl-*N*^1^-feruloylspermidine **42** and *N*^5^-*p*-coumaroyl-*N*^1^,*N*^10^-diferuloylspermidine **43** were named keayanidines A and B [64] (Figure 9). The new compounds were extracted with MeOH from defatted *M. keayana* roots at room temperature. After column chromatography followed by reversed-phase HPLC, it was possible to isolate keayanidines A and B. Their complete structures were elucidated using positive-mode electrospray mass spectrometry/mass spectrometry (ESI-MS/MS) and ^1^H- and ^13^C-NMR spectroscopy.

Compound **44** was isolated from an 80% aqueous methanolic extract of pollen from *Quercus dentata* and information on the position of the hydroxycinnamoyl moieties was derived from electrospray mass spectral fragmentation patterns [65,66] (Figure 9).

**Figure 10:**
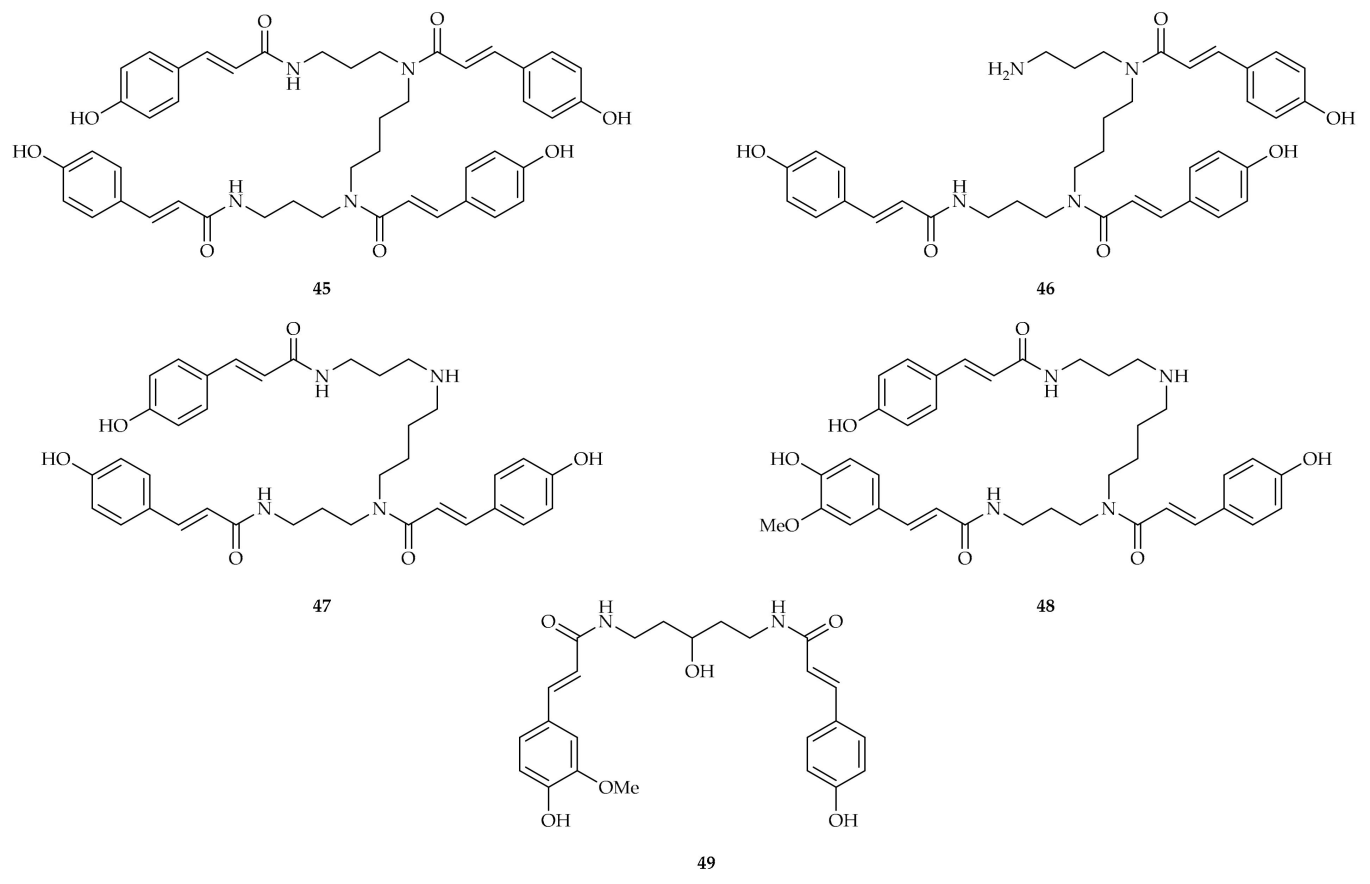
Chemical structures of *N*^1^,*N*^5^,*N*^10^,*N*^14^-tetra-*p*-coumaroylspermine **45**, *N*^1^,*N*^5^,*N*^10^- tri-*p*-coumaroylspermine **46**, *N*^1^,*N*^5^,*N*^14—^tri-*p*-coumaroylspermine **47** (keayanine B), *N*^1^-feruloyl-*N*^5^,*N*^14^-di-*p*-coumaroylspermine **48** (keayanine C), and *N*-*trans*-*p*-coumaroyl-*N*′-*trans*-feruloyl-3-hydroxycadaverine **49**.

Compounds **45** and **46** were isolated from dried flowers of *Matricaria chamomilla* L. (chamomile) and characterized [67] (Figure 10). Elucidation of their structures was conducted by means of NMR spectroscopy and ESI-LC-MS analysis.

Tetra-coumaroylspermine **45** is also an active component in *Coreopsis tinctoria*, known in China as “snow chrysanthemum”, used to prepare a flower tea with antioxidant and antidiabetic activities [68]. The compound is also present in Xiaoheiyao, the rhizome of *Inula nervosa* Wall., a spice used in Chinese food preparations. It has been demonstrated that beef patties marinated with the spice produce lower amounts of carcinogenic aromatic heterocyclic amines when grilled, in comparison to untreated meat. This is due to the electrophilic capture of the precursor creatinine by the *p*-coumaroyl derivative [69].

The following spermine derivatives were identified in *Microdesmis keayana* and *Microdesmis puberula*: *N*^1^,*N*^5^,*N*^14^-tri-*p*-coumaroylspermine **47** and *N*^1^-feruloyl-*N*^5^,*N*^14^-di-*p*-coumaroylspermine **48**, named keayanines B and C, respectively. These plants are used in traditional central African medicine [64,66] (Figure 10).

*p*-Coumaroyl-feruloyl-3-hydroxycadaverine **49** was isolated and characterized by NMR from *Alisma orientalis* (Sam.) Juzep. (known in Chinese as “Ze-Xie”) (Alismataceae), comprising approximately 11 species widely distributed in temperate and subtropical regions [70] (Figure 10).

**Figure 11:**
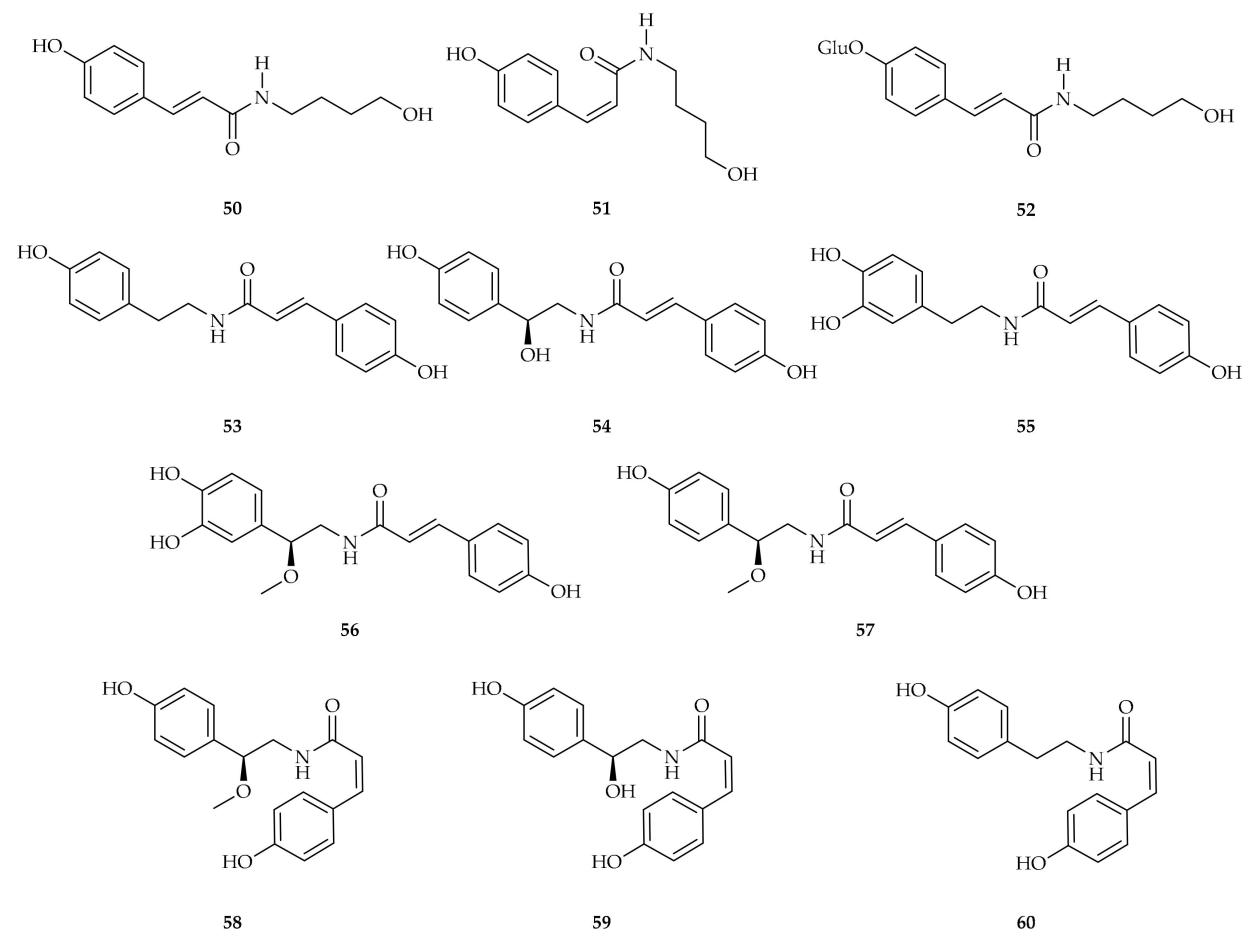
Chemical structures of 4-[(*E*)-*p*-coumaroylamino]butan-1-ol **50**, 4-[(*Z*)-*p*-coumaroylamino]butan-1-ol **51**, compound **52**, *p*-coumaroyltyramine **53** (paprazine), *p*-coumaroyloctopamine **54**, *p*-coumaroyl-3′,4′-dihydroxyphenylethylamine **55**, phenylethylamine A **56**, compound **57**, compound **58**, *cis-p*-coumaroyloctopamine **59**, and *cis*-*p*-coumaroyltyramine **60**.

4-[(*E*)-*p*-Coumaroylamino]butan-1-ol **50** and 4-[(*Z*)-*p*-coumaroylamino]butan-1-ol **51** were first isolated from *Hippophae rhamnoides* L. subsp. *sinensis* Rousi (Elaeagnaceae) by Wang et al. in 2015 [71] (Figure 11). This deciduous shrub, also known as seabuckthorn, is mainly present in Europe and Asia and its fruits are rich in vitamin C, vitamin E, carotenoids, flavonoids, and health-beneficial fatty acids. The two compounds were isolated via AcOEt and BuOH extraction of the seed residue of the fruits, and their structures were determined using 1D and 2D NMR techniques.

Compound **50** was also isolated from *Cannabis sativa* L. together with its glucoside compound **52** [72] (Figure 11).

*p*-Coumaroyltyramine **53**, commonly known as paprazine, together with other *N*-(hydroxycinnamoyl)tyramines, is synthesized in plants for cell wall fortification in response to *Phytophthora* infestans attack. The synthesis of *N*-(hydroxycinnamoyl)tyramines is catalyzed by hydroxycinnamoyl-CoA/tyramine hydroxycinnamoyl transferase (THT). Since paprazine (*p*-coumaroyltyramine) and its stereoisomer *cis*-*p*-coumaroyltyramine were the topic of a very recent review [73], only similar compounds are described in the present section (Figure 11).

*p*-Coumaroyloctopamine **54** was first isolated from the roots of eggplant (*Solanum melongena* L.) in 1978 [74]. Later on, this compound was isolated from other plants, and it was established to also contribute to antioxidant activity [75] (Figure 11).

Compound **54**, together with other *p*-coumaroyl amides (compounds **55**–**60**), were found in the leaves of *Solanum rostratum*, a malignant invasive weed (Figure 11) [76]. This exotic plant exhibits an invasive mechanism as a defensive mechanism against herbivorous insects. All these compounds have been tested for their antifeedant activity against *Helicoverpa armigera*. Compounds **55** and **56** displayed potent antifeedant activities, with an antifeedant index (AI) of 65.1 and 61.6% at 100 μM, respectively. Compound **56** was also found in other Solanaceae species, such as *Solanum melongena*. Furthermore, compound **57** was found in *Tribulus terrestris* L, a traditional medicine for treating headaches, high blood pressure, dizziness, menstrual irregularities, sexual dysfunction, and cardiovascular risk [77]. In addition, compounds **57** and **58** were found in *N. physaloides*, a plant rich in phenolamides that displays many health properties, particularly against memory impairment [78].

**Figure 12:**
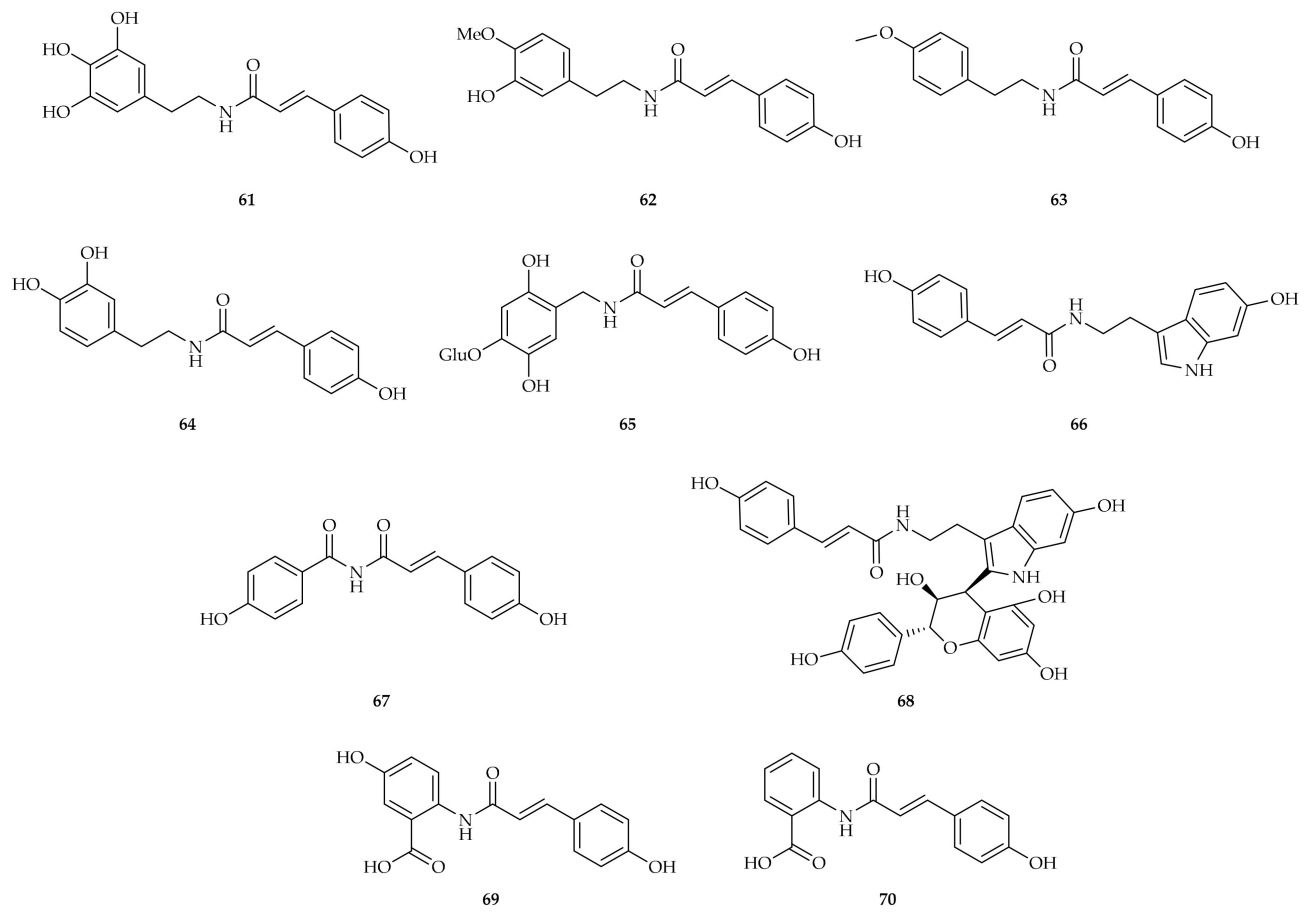
Chemical structures of compounds **61**–**63**, *p-*coumaroyldopamine **64**, oleraciamide E **65**, hygarine **66**, tribulusimide C **67**, lotthanongine **68**, avenanthramide A **69**, and avenanthramide D **70**.

In 2012, Hu and colleagues isolated compounds **61** and **62** from *Microlepia pilosissima*; their structures were assigned by NMR spectroscopy and MS [79] (Figure 12).

Similar amides have been isolated from *Capsicum annum*, particularly compound **63**, whose structure was determined using NMR spectroscopy [80] (Figure 12).

*p-*Coumaroyldopamine **64** was detected and isolated for the first time after bacterial infection of tomato plants (*Solanum lycopersicum* cv. Rutgers) with *Pseudomonas syringae* pv. Tomato indicating again that these compounds could play a defensive role against bacterial infection in plants (Figure 12). The accumulation of hydroxycinnamoylamides was preceded by an increase in hydroxycinnamoyl-CoA/tyramine *N*-hydroxycinnamoyl transferase (THT) gene expression [81].

One novel *p*-coumaroyl amide glycoside, named oleraciamide E **65**, was isolated from *Portulaca oleracea* L. [82] (Figure 12).

An indole compound derivative of *p*-coumaric acid is hygarine **66**, so called because of the plant in which it was found, *Hygroryza aristata* (Gramineae), a perennial plant distributed in tropical Asia [83] (Figure 12).

Tribulusimide C **67**, a new cinnamic imide derivative, was isolated from the fruits of *Tribulus terrestris* [84], while lotthanongine **68**, a novel flavonoidal indole alkaloid, was isolated from the roots of *Trigonostemon reidioides* (Euphorbiaceae) (Figure 12). The aqueous extract of the roots causes vomiting and is used in Thai traditional medicine as an antidote for detoxification of poisonous mushrooms, as well as externally for antiseptic purposes [85].

Avenanthramides A **69** and D **70** are part of the class of compounds named avenanthramides (AVNs), which are unique to oats of the genus *Avena* (Poaceae) [86] (Figure 12).

### 3.2. Biological Activity

#### 3.2.1. Antioxidant, Anti-Inflammatory, and Cardiovascular Activities

Cocoa beans (*Theobroma cacao* L.) and cocoa liquor are rich sources of *N*-phenylpropenoyl-L-amino acids (NPAs). The quantity of the NPAs depends on the developmental stage of cocoa seeds. Ripe cocoa pods, the raw materials for chocolate production, go through fermentation for 6–8 days, acquiring the characteristic brown color with a change in the composition of the secondary metabolites. For example, in 2013, Gema Pereira-Caro et al. analyzed the Trinitario cultivar of *Theobroma cacao* at different growth stages and NPA levels were higher in the final stage of cocoa bean development. Among the identified cinnamoylamino acids, *p*-coumaroyltyrosine **5**, *p*-coumaroylaspartate **6** and caffeoylaspartate were predominant [87]. These compounds, along with *p*-coumaroylglutamate **7** and other caffeoyl and feruoyl amino acids, were previously detected in fresh cocoa seeds [88]. *p-*Coumaroyltyrosine **5** is also present in cocoa calli, a type of tissue that is formed when plants are injured or damaged. Because of its antioxidant nature, the abundant synthesis of compound **5** could be a defensive reaction against stress [89,90]. Furthermore, NPAs are important antioxidants for preventing lipid peroxidation and protecting cellular membranes from oxidative damage. Since NPAs have high affinities for membranes, they can be integrated into membranes to inhibit peroxidation and prevent chronic diseases due to oxidative stress, such as neurological diseases or atherosclerosis [91]. Comparative analysis of antioxidant capacity using the DPPH assay demonstrated that caffeoylamino acids have the highest scavenging activity, followed by feruloyl- and *p*-coumaroylamino acids. The phenolic ring has been found to play a crucial role in the antioxidant activity of hydroxycinnamoyl amino acids because it can donate an electron to free radicals and neutralize them, transforming itself into a phenoxy radical. The hydroxyl group in the *ortho*-position in caffeoylamino acids facilitates transfer of a hydrogen atom to the free radicals. Furthermore, the presence of the hydroxyl group stabilizes the phenoxy radical through intramolecular hydrogen bonds, thus enabling the formation of the *ortho*-quinone compound [28]. The methoxy group in feruloyl amino acids can also facilitate the neutralization of free radicals; however, it cannot form *ortho*-quinone compounds, resulting in reduced antioxidant ability. Conversely, *p*-coumaroylamino acids are the least powerful radical scavengers due to the absence of an electron-donating group at the *ortho*-position [91].

Abrusamide A **15**, abrusamide D, and abrusamide H were investigated as inhibitors of nitric oxide (NO) production in lipopolysaccharide (LPS)-induced RAW264.7 macrophages. They showed potent inhibitory activities without influencing cell viability, with IC_50_ values of 23.4, 25.2, and 28.3 μM, respectively. Aminoguanidine (AG) was used as the positive control. Abrusamide A **15** also exhibited hepatoprotective effects against human L-02 cells [27]. A fraction of the *Rosa rugose* extract contained compound **34,** perhaps the most interesting *p*-coumaroyl amide due to its biological activity, exhibiting remarkable hepatoprotective activity with a similar effective dosage to sulforaphane. The hepatoprotective effect of compound **34** against EtOH exposure was further evaluated using HepG2 cells. Compound **33** showed no cytotoxicity against HepG2 cells when its concentration was lower than 100 μM (24 h); however, it significantly increased cell proliferation at a concentration ≥ 200 μM (24 h) [92].

A recent study demonstrated that the intake of *p*-coumaroylserotonin **16** at a dose of 7.5 mg kg^−1^ day^−1^ led to a decrease in oxidative stress, reducing ROS levels and regulating the mitogen-activated protein kinase pathway in cisplatin-induced renal tissue mice [93,94].

The antioxidant activity of compound **16** has an important impact on its activity against cardiovascular risk and diseases. It has a strong inhibitory activity on the oxidation of low-density lipoproteins (LDLs), and a dietary supplement of this compound in mice reduced the triglyceride levels in plasma by 10% and the level of LDLs by 35%, while enhancing the level of HDLs by 25%. The effect was more pronounced when a combination of compound **16** and *p*-feruloylserotonin was used, while the glycosides were less effective. Serotonin derivatives like *p*-coumaroylserotonin also have hypocholesterolemic effects and increase the ratio of HDL/total cholesterol in ovariectomized rats because estrogens can influence HDL and LDL levels in the blood, and thus the lipid status [95].

*p*-Coumaroylserotonin **16** also plays a role in atherosclerosis. Reduction of atherosclerosis in the aortic sinus of Apo-E-deficient mice was evaluated; results showed high reductions when the pure compound was used (up to 53% reduction in the lesioned areas), and even higher (79%) when safflower seed extracts containing a comparable amount of the serotonin amides was used [96].

Proinflammatory cells inside the vascular wall can produce a large amount of reactive oxygen species, leading to low-density lipoprotein (LDL) oxidation and consequently foam cell deposition, causing atherosclerosis. Koyama and colleagues demonstrated that ethanol–ethyl acetate extract of defatted safflower seeds inhibited LDL oxidation induced by V70, a free-radical-generating system, and by CuSO_4_, a metal-ion-dependent lipid peroxidation system. Furthermore, the synthetic compound **16** showed significant inhibition of in vitro-induced LDL oxidation and reduced the sensitivity of plasma to oxidation ex vivo [97]. Another cause of atherosclerosis is the migration of vascular smooth muscle cells (VSMCs) into intima or their excessive proliferation in the neointima. The free cytosolic concentration of calcium ions and the platelet-derived growth factor (PDGF) are involved in modulating VSMC movement and proliferation. Compound **16** inhibited the release of free Ca^2+^ in the cytosol of rat VSMCs induced by KCl or 5-hydroxytryptamine and it also inhibited PDGF-BB in a concentration-dependent manner. As a result, *p*-coumaroylserotonin **16** could block the activation of VCMNs via inhibition of calcium ion release or by inactivating PDGF signaling [98].

In another study, Katsuda and his group investigated the significant contribution of this compound to reducing cardiovascular risk and ameliorating wall distensibility. They measured the arterial stiffness of a group of Kusanagi-hypercholesterolemic (KHC) rabbits treated with serotonin derivatives like *p*-coumaroylserotonin **16**, and the results demonstrated inhibited progress of atherosclerosis and improvement of wall distensibility [99].

Furthermore, research has also demonstrated that this compound has the potential to increase the number of fibroblast cells in human and mouse models. In their study, Takii and colleagues tested the proliferation of fibroblasts in the presence of compound **16**, demonstrating a significant increase in fibroblast cells without any accompanying increase in other cell types, particularly in the absence of tumor cells. Consequently, as compound **16** is common in safflower seeds and cake oil, the daily intake of safflower oil can stimulate the repair of damaged tissues. However, the same administration could be harmful because of the increase in fibroblast cells, which could lead to fibrosis. It is an issue to be considered but it is possible to conclude that compound **16** is a tool that should be investigated to determine its regulatory mechanism in fibroblast cell proliferation [100].

*p*-Coumaroylserotonin **16** also inhibits the migration of monocytes to endothelial cells and the transcription of NF-kB [101]. However, the proinflammatory process is also activated by LPS-stimulated human blood monocytes and compound **16** can also inhibit NF-kB activation induced by this toxin [102]. Furthermore, in 2000, Nagatsu and colleagues discovered *p*-coumaroylserotonin **16**’s potential as an inhibitor of Epstein–Barr virus’s early antigen activation induced by phorbol-12-myristate-13-acetate [103].

In addition, compound **16** attenuates osteoarthritis (OA), a degenerative condition causing cartilage damage, pain, swelling, and joint stiffness. Pathological cartilage destruction in osteoarthritis involves upregulation of catabolic factors such as metalloproteinases (MMPs), a disintegrin and metalloproteinase with thrombospondin motifs 5 (ADAMTS5), and activation of the NF-kB pathway. *p*-Coumaroylserotonin **16** attenuated cartilage damage, reducing the levels of MMPs and ADAMTS5 and blocking NF-kB factor signaling via inhibition of IκB degradation and p65 phosphorylation [104].

Diferuloylputrescine and *p-*coumaroylferuloylputrescine **25** exhibit antioxidant activities [105]. (*E,E*)-di-*p*-coumaroylputrescine **24** exhibited the strongest hydroxyl radical scavenging activity (IC_50_ 120.55 µM) using the DPPH assay [45]. Compounds **42** and **43** showed radical scavenging activity, as measured by the DPPH assay, giving IC_50_ values of 25.3 and 27.9 μM. The two keayanidines are thus weaker scavengers in comparison to quercetin (IC_50_ of 8.3 μM) [64]. Compound **49** exhibited the most promising activity in protecting H₂O₂-induced damage in SH-SY5Y cells, which are human dopaminergic neuroblastoma cells. Despite testing a range of caffeoyl and feruloyl polyamides with different configurations (*trans* or *cis*), only compound **49** demonstrated the most effective protection against oxidative stress. Given that this compound only contains a *p*-coumaroyl moiety, it suggests that the configuration of hydroxycinnamic moieties is a more significant factor than the phenolic ring’s substitution [70]. Also, AVNs were shown to possess anti-inflammatory properties, and high levels of AVNs in the diet suppress high-fat-diet-induced atherosclerosis in a mouse model [86].

Compounds **50** and **52** inhibited TNF-α release from LPS-induced BV2 microglia cells, suggesting that they can downregulate the LPS-mediated production of inflammatory molecules to protect cells from inflammation irritation. This is especially true for compound **52** (TNF-α = 1644.68 pg/mL compared with resveratrol, the positive control, TNF-α = 1479.03 pg/mL) [72].

#### 3.2.2. Anticancer Activity

*p-*Coumaroylserotonin **16**, isolated from the seeds of *Centaurea vlachorum*, showed a promising role in the treatment of glioblastoma as it can cross the blood–brain barrier (BBB). Compound **16** decreases cell viability, inducing cell cycle arrest at S phase and apoptosis and increasing caspase-8 activity [106]. It also exhibits a cytotoxic and antiproliferative effect in lung cancer, causing the arrest of S phase and decreasing the expression of CD15/CD56/CD24/CD44/CD58/CD71, proteins that increase the mobility of tumor cells. Compound **16** isolated from *Croton echioides* is also active in vitro against the human colon cancer line HCT-116. In addition, it is also active against Henrietta Lacks cervical cancer (HeLa) and Michigan Cancer Foundation-7 (MCF-7) breast cancer cell lines [32,35].

Compounds **27** and **28** exhibit cytotoxicity against A549, SK-OV-3, SK-MEL-2, and HCT-15 human cancer cells [53].

Compound **32**, isolated from defatted adlay, showed great anticancer ability on HepG-2, MCF-7, and CaCo-2 cancer cell lines by MTT assays, with IC_50_ values between 46.34 ± 3.99 and 92.69 ± 5.63 μg/mL [107]. Furthermore, Wang et al. showed that compound **32** can protect HepG2 cells from oxidative stress by increasing the antioxidant enzymes regulated by Nrf2/ARE pathway [108].

Recently, it was demonstrated that the combination of avenanthramide A (AVN A) **69** and 5-fluorouracil (5-FU) has significant therapeutic advantages against colorectal cancer (CRC). Moreover, AVN A **69** mitigated the systemic adverse effects of 5-FU [109].

#### 3.2.3. Antidiabetic Activity

*p*-Coumaroylserine **9** (Figure 3) was identified in 2021 in the ethanolic extract of *Launaea nudicaulis* (L.) Hooker fil. (Asteraceae), native to an area of Spain in the west through to North Africa, the Arabian Peninsula, and western Asia to India in the east [110].

The extract was evaluated on diabetic complications in streptozotocin-induced hyperglycemic rats. A protective effect on the pancreas, liver, kidney, and testis that degenerated in diabetic control rats was observed. This effect cannot be directly attributed to compound **9** since 85 different compounds were identified in the extract.

It is postulated that the activity is a synergized action and fractionation of the crude extract and isolation of pure compounds is necessary to confirm the antidiabetic activity of compound **9**.

α-Glucosidase is an enzyme involved in carbohydrate metabolism, resulting in an optimal target to avoid hyperglycemia, bodyweight changes, and mortality associated with diabetes mellitus. Diabetes mellitus causes high blood glucose levels and several complications such as cardiovascular problems, neuropathy, and cancer. The management of diabetes, by controlling glucose levels in blood, is a good way to reduce the risk of developing cancer [111]. *p*-Coumaroylamino acids offer an alternative to synthetic drugs such as acarbose, which has many side effects, including flatulence, abdominal pain, and diarrhea [112]. The ethanol extract of *Abutilon fruticosum* (Malvaceae) exhibits strong inhibitory activity. *Abutilon fruticosum* is native to Africa, southern and southwestern Asia, northern Mexico, and the south–central United States. A bioassay-guided fractionation of *A. fruticosum* extracts as potential AGIs and cytotoxic agents was performed. The activity of extracted phytochemicals was tested using in silico pharmacophore and docking studies. Comparing butanol extract, acarbose, and ethanol extract, the best inhibitory activity was given by the ethanol extract from which it was possible to isolate *p*-coumaroyltyrosine **5,** which gave the highest docking score and showed the best interaction with the binding pocket of α-glucosidase.

In 2022, Manh Tuan Ha et al. extracted *p*-coumaroyltryptophan **8,** *p*-coumaroyltyrosine methyl ester **12**, and *p*-coumaroyltryptophan methyl ester **14** from fruits of *Hedera rhombea*, a medicinal plant used in folk medicine in many countries. *p*-coumaroyltyrosine methyl ester **12** displayed significant inhibitory activity at 82.73 μM, stronger than the positive control (acarbose, IC_50_ = 298.07 μM), while *p*-coumaroyltryptophan **8** and its methyl ester **14** showed lower inhibitory activities compared to the caffeoyl moiety, suggesting the importance of the caffeoyl moiety in L-tryptophan derivatives [113].

Although compound **16** showed a lower inhibitory activity against α-glucosidase with respect to the reference compounds acarbose and 1-deoxynojirimycin [114], Takahashi and colleagues determined that the hydroxyl group in position 5 is essential for inhibition. They also evaluated the inhibitory activity of *p*-coumaric acid to clarify the role of the *p*-coumaric moiety. It did not exhibit any inhibitory activity, indicating that both the free hydroxylic group in position 5 and the amide bond with the indole system are important [115]. In 2015, Seo and colleagues confirmed the potential of compound **16** as inhibitor of α-glucosidase, reporting also the significant inhibition of mammalian rat intestinal sucrase and the reduced glucose level in human intestinal epithelial cells [116].

di-*p*-Coumaroylputrescine **24** and feruloyl-coumaroylputrescine **25** have been identified as highly effective inhibitors of the alpha-glucosidase enzyme [117]. It was demonstrated that it is necessary for at least one *p*-coumaroyl moiety to have good inhibitory activity [118].

In vitro and in vivo studies of the anti-hypoglycemic effects and mechanisms of action of *p*-coumaroyloctopamine **54** have recently suggested that it exerts anti-hypoglycemic and antioxidant effects, most probably via the regulation of a PI3K/AKT/GSK3β signaling pathway [119].

#### 3.2.4. Melanogenesis

Tyrosinase is the enzyme responsible for the biosynthesis of melanin in plants and animals. When it is overactive, it plays a role in hyperpigmentation and skin disorders, such as melanoma and melasma. Due to this, there is increasing research focused on the development of tyrosinase inhibitors. Natural polyphenols such as *p*-coumaroyl amino acids have been investigated as possible tyrosinase inhibitors. Testing caffeoyl-, feruloyl-, and *p*-coumaroyl amino acids on mushroom tyrosinase have shown better activity of *p*-coumaroyl amino acids compared to caffeoyl- and feruloylamino acids. It seems that the addition of a hydroxy or a methoxy group to the *p*-hydroxyphenyl moiety leads to a decrease in anti-tyrosinase activity. *p*-Coumaroyl-DOPA **11** followed by *p*-coumaroyltyrosine **5** displayed significant inhibitory activities but remained less potent than the standard inhibitor, hydroquinone [120].

The 80% aqueous methanol extract and ethyl acetate fraction from safflower seeds (*Carthamus tinctorius* L.) were also evaluated for their melanogenesis inhibitory activity; significant inhibition of mushroom tyrosinase was observed. Two hydroxycinnamoylserotonine derivatives, feruloylserotonin and *p*-coumaroylserotonin **16,** are among the active compounds. They were isolated from the ethyl acetate fraction and showed IC_50_ values of 0.023 and 0.074 mM, respectively, compared with arbutin (IC_50_ 0.223 mM) as the reference compound. Moreover, compound **16** strongly inhibited melanin production by *Streptomyces bikiniensis* and B16 melanoma cells [30]. The structure of compound **16** was investigated by docking analysis, suggesting a good fit in the binding pocket of tyrosinase because it can form hydrogen bonds with amino acid residues in the pocket, compatible with the volume of the molecule [121].

Compound **24** showed tyrosinase inhibitory activity toward L-tyrosine as the substrate (IC_50_ 181.73 µM). The ability to inhibit melanin synthesis in B16 melanoma cells was also studied, with compounds **24** and **25** again demonstrating the most effective IC_50_ values. This indicates that the coumaric moiety plays a crucial role in melanin synthesis inhibition. The results are significant as they demonstrate the potential of polyamine conjugates, which are naturally occurring, as scavengers of free radicals that can damage human tissues. Also, compounds **39**–**41** are inhibitors of tyrosinase, but the most powerful compounds were those with *p*-coumaroyl moieties [45].

#### 3.2.5. Neurological Effects

Compound **16** was effective in ameliorating memory in scopalamine-induced AD mice, down regulating acetylcholine esterase activity. It exhibited in vitro acetylcholine esterase inhibitory activity with an IC_50_ of 15 μg mL^−1^ and an inhibitory activity with an IC_50_ value of 1.2 μM on monoamine oxidase B, a mitochondrial outer membrane enzyme correlated with aberrant Aβ production in neurons [33]. Furthermore, this compound also inhibited β-secretase-1, the major enzyme in the production of amyloid beta-peptides that aggregate in the brain of Alzheimer’s patients. In addition, compound **16** was active as an inhibitor of AChE, with 72.6% inhibition and an IC_50_ value of 15.0 μg/mL. Based on this evidence, compound **16** could be considered a promising compound for treating AD.

The extract of *Aphelandra tetraffona* and *A. chamissoniana* with deep eutectic solvents, containing compounds **32**–**36**, acted as an acetylcholinesterase inhibitor, with an IC_50_ of 714 μg/mL, a lower value than conventional solvent foxtail millet bran extract (831 μg/mL). The higher levels of hydroxycinnamic acid amides extracted suggest that they can contribute to the anti-acetylcholinesterase activity [60]. Compound **30** has been shown to block chemical synaptic transmission at invertebrate neuromuscular junctions, with an IC_50_ of approximately 200 μM [122]. Compound **45** inhibited the contraction of the guinea pig ileum and exhibited antagonism towards NK1 receptor, with K_i_ values of 21.9 nM and 3.3 nM, respectively, while compound **46** did not exhibit any tachykinin antagonist activity [67].

Compounds **55** and **56** displayed marked in vitro inhibitory effects on both AchE and CarE (EC_50_ = 16.87 ± 0.83 and 23.14 ± 1.54 μM and 41.29 ± 1.54 and 46.87 ± 0.83 μM, respectively) and interacted with AchE at Gly187 and CarE at Ala235, Tpp342, and Phe395, as revealed by molecular docking and molecular dynamic simulation studies [76].

Compound **65** exhibited anticholinesterase activity, with an IC_50_ value of 52.43  ±  0.33 μM. Moreover, it showed scavenging activity in the 1,1-diphenyl-2-picrylhydrazyl (DPPH) radical quenching assay, with an IC_50_ value of 24.64  ±  0.33 μM [82].

Recently, *p*-coumaroylspermidine extract containing compound **34** and compounds **38**–**40** was shown to have a significant antidepressant effect, as demonstrated using a chronic unpredictable mild stress (CUMS) rat model in which the compounds were observed to inhibit the increase in the levels of corticosterone and the decrease in levels of 5-hydroxytryptamine, dopamine, and noradrenaline induced by CUMS [62].

Compound **38** potently and selectively inhibited serotonin uptake in S6 cells or in synaptosomes, with IC_50_ of 0.74 ± 0.15 µM for S6 cells and 1.07 ± 0.23 µM for synaptosomes, and a reversible competitive property for 5HT uptake inhibition. Its potency for 5HT uptake was weaker than that of fluoxetine, whereas its efficacy was generally similar [63].

#### 3.2.6. Antiviral Activity

Compound **34** was extracted from *Artemisia caruifolia* with methanol and tested for HIV-1 protease inhibition. The results showed significant inhibitory activity, with an IC_50_ of 53 μg/mL [44]. The same researchers synthesized analogues of this compound in order to enhance the inhibition; they found that *N*^1^, *N*^5^,*N*^10^,*N*^14^-tetra-*p*-coumaroylspermine and *N*^1^,*N*^4^,*N*^7^,*N*^10^,*N*^13^-penta-*p*-coumaroyltetraethylenepentamine showed more potent activity, suggesting that hydroxyl groups influence the inhibition, and the effect is abolished when they are acetylated. Furthermore, analogues with the same number of amide bonds and shorter chains showed no inhibitory activity, suggesting that even chains influence inhibitory activity. This information provides the basis for further structural modifications of compound **34** to find potent anti-HIV agents. Compound **46** also showed a modest inhibitory activity against HIV-1 protease (IC_50_ of 83 μM) [44].

#### 3.2.7. Antimicrobial Activity

*p*-Coumaroyltyrosine **5** has been identified in *A. fistulosum* L., (Amaryllidaceae) (ex-Alliaceae), commonly known as Welsh onion, which has a similar taste and smell to common onion. Zolfaghari et al. investigated the potential of this compound as an antibacterial against *E. coli* and *Staphylococcus aureus*. The results showed that compound **5** was active against *S. aureus* but not against *E. coli* [123].

#### 3.2.8. Anthelmintic Activity

Abdel-Moez et al. isolated some NPAs from *Amaranthus blitum* L., a plant used for treating intestinal disorders, roundworm infections, and hemorrhage [124]. In their work, the anthelmintic activities of the plant extracts against *Trichinella spiralis* were evaluated to determine which compounds were effective. Both *trans*- and *cis*-*p*-coumaroyltryptophan, compounds **8** and **10**, respectively, were isolated and were found to completely induce larval death within 48 h. The indole heterocycle of tryptophan could be the reason of this successful activity; in fact, previous papers have shown the presence of an indole moiety in the structure of many anthelmintics [125,126]. However, to gain complete knowledge, it is necessary to study the relationship between the good anthelmintic activity and the structure of this class of compounds.

Compound **27** was also shown to have anthelmintic activity against *Dactylogyrus intermedius* in goldfish (*Carassius auratus*), with an EC_50_ of 1.4 mg/L and acute toxicity LC_50_ of 7.4 mg/L [127].

Compound **54**, despite its similarity to paprazine, was less active, probably due to the presence of the hydroxyl group showing moderate inhibition (MIC 64 mg/mL) against Gram-positive (*S. aureus* ATCC25923) and Gram-negative (*K. pneumoniae* ATCC11296) bacterial strains [128].

#### 3.2.9. Other Activities

Since the highest quantity of *p*-coumaroylamino acids is present in cocoa nibs and cocoa liquor, and *p*-coumaroyltryptophan **8** is quite abundant in raw Robusta coffee beans, their bioavailability has been studied. In 2008, Stark et al. administered a cocoa drink containing defined amounts of NPAs to healthy volunteers and following the urinary excretion of these amides over time using stable isotope dilution assay (SIDA) with LC-MS/MS multiple reaction monitoring (MRM) detection [129]. The highest quantity was found for caffeoylaspartic acid and *p*-coumaroylaspartic acid **6**, followed by *p*-coumaroyltyrosine **5** and caffeoyl-3-hydroxytyrosine. The *p*-coumaroylamino acids, particularly *p*-coumaroylaspartic acid **6**, *p*-coumaroylglutamic acid **7**, and *p*-coumaroyltyrosine **5**, were the main NPAs in urine 2 h after administration, while caffeoylaspartate was not present. This bioavailability study emphasizes the non-absorption of *p*-coumaroyl amino acids, excluding their metabolic transformation in the human body.

*Aciculosporium take* (Ascomycota; Clavicipitaceae) causes witches’ broom disease in bamboo, particularly *Phyllostachys bambusoides*. In order to understand the development of this disease, researchers focused on the physiological changes in affected bamboo, discovering low levels of auxin (indole-3-acetic acid), suggesting that it is necessary to investigate the indolic compounds present in infected bamboo [130].

Compound **18**, isolated from safflower seed, showed anti-osteoporosis effects in a zebrafish model [131].

The antifungal activity of *N*^1^,*N*^5^-di-*p*-coumaroyl-caffeoylspermidine **44** was determined against *Pyrenophora avenae* and *Blumeria graminis*. It reduced mycelial growth and powdery mildew when applied as a post-inoculation treatment. Growth of *P. avenae* in the presence of 100 μM of compound **44** reduced the activity of S-adenosylmethionine decarboxylase (AdoMetDC) and led to a reduction in the incorporation of labelled ornithine into spermidine [65].

Diferuloylputrescine and *p-*coumaroylferuloylputrescine **25** inhibited aflatoxin B1 biosynthesis in *Aspergillus flavus* [132]. The inhibition was shown to be concentration-dependent and could reach 93%.

## 4. Tertiary Amides

### 4.1. Isolation, Characterization, and Role in Plants

**Figure 13:**
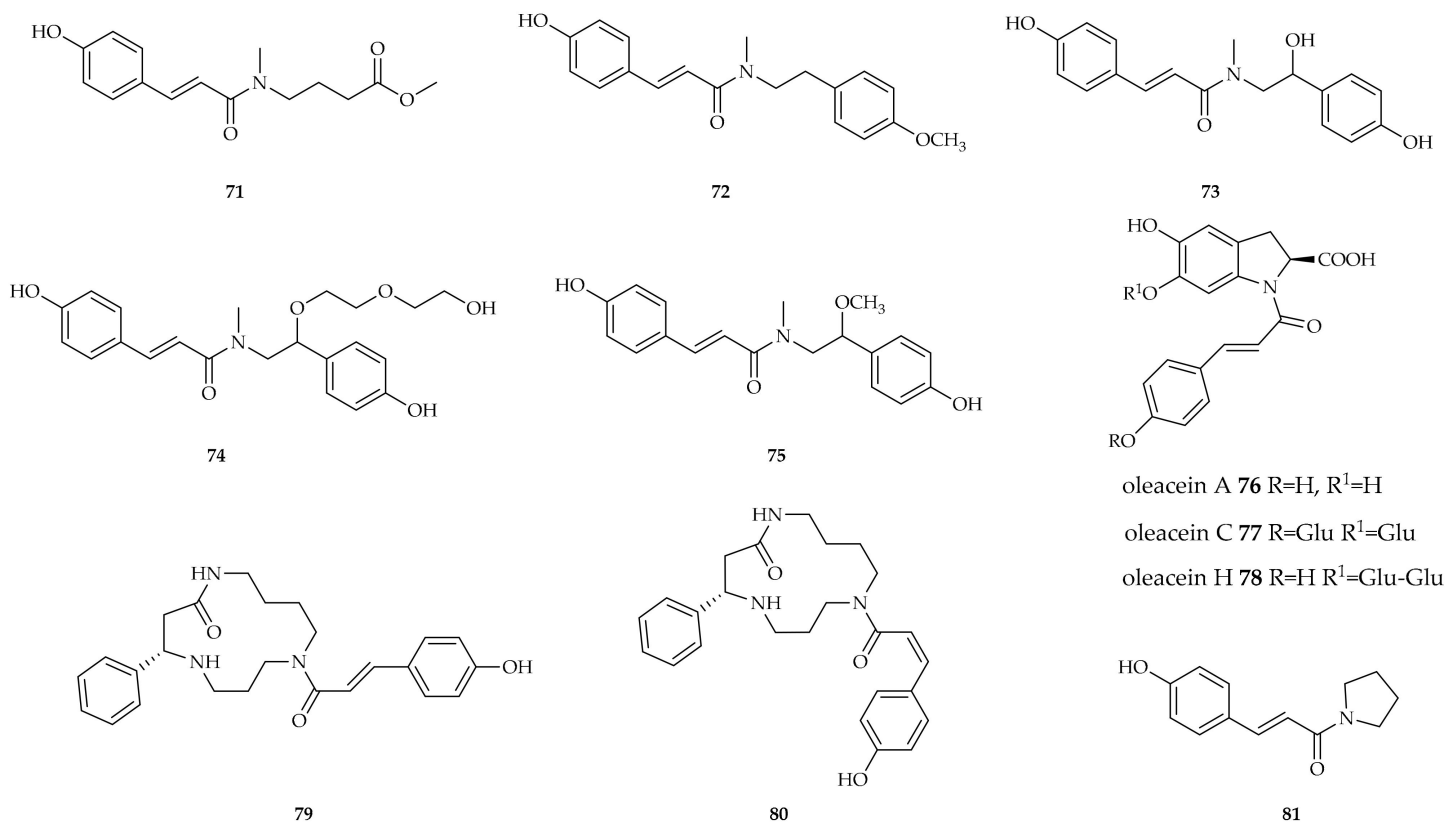
Chemical structures of baimantuoluoamide A **71**, ailanthamide **72**, micrometam A **73**, micrometam B **74,** micrometam E **75**, oleraceins **76**–**78**, meefarnines A **79**, meefarnines B **80,** and piperlotine E **81**.

Compound **71**, also named baimantuoluoamide A, was isolated in 2010 by Kuang et al. [133] from the flowers of *Datura metel* L., an annual herb belonging to the family Solanaceae, used for the treatment of asthma, cough, convulsion, and insanity in China. This new alkaloid was isolated from the alkaloidal fraction of the dried flowers (Figure 13).

The new amide ailanthamide **72** was isolated in 2009 by Chen et al. [134] from *Zanthoxylum ailanthoides* Sieb. & Zucc. (Rutaceae), a medium-to-large-sized tree found at low altitudes in forests in China, Korea, Japan, the Philippines, and Taiwan. Its leaves are used as a folk medicine to treat the common cold in Taiwan (Figure 13).

The methyl amides micrometam A **73**, micrometam B **74,** and micrometam E **75** were isolated in 2014 by Luo et al. from *Micromelum falcatum* (Lour.) Tanaka, a shrub widely distributed in southeastern Asia [135] (Figure 13).

Oleraceins are indoline alkaloids typically found in common purslane (*Portulaca oleracea L.*), a plant widely used both as food and in many traditional medicines. Several oleraceins are amides of phenolic acids, and oleracein A **76** is a *p*-coumaroyl amide, while oleracein C **77** and H **78** are glucoside derivatives of oleracein A [136] (Figure 13).

Meefarnines A **79** and B **80** are cyclic spermidine amides first isolated from *Meehania fargesii*, a plant used as an antipyretic in Chinese medicine [137] (Figure 13).

Compound **80** was also identified in a combined extract from *Lithospermum erythrorhizon*, *Houttuynia cordata*, and *Spirodela polyrhiza* [138] (Figure 13).

Compound **81**, named piperlotine E, was isolated in 2007 by Wu et al. from *Piper lolot* (Piperaceae) [139], a small shrub found widely at lower elevations in Vietnam and used to treat various diseases, such as rheumatism, lumbago, digestive troubles, vomiting, and diarrhoea (Figure 13).

### 4.2. Biological Activity

Compound **72** showed inhibition of superoxide anion generation by human neutrophils in response to formyl-L-methionyl-L-leucyl-L-phenylalanine/cytochalasin B (fMLP/CB) and inhibition of MLP/CB-induced elastase release, with IC_50_ values of 3.71 and 4.23 µg/mL, respectively [134].

Despite the fact that many beneficial activities have been claimed for purslane extracts, including actions on neurodegenerative diseases [140], evidence in the literature is scarce, and oleracein A **76** and its glucosylated derivatives have been tested only for their antioxidant activity [141].

Compound **80** showed strong activity in alleviating atopic dermatitis [138].

Compound **81** inhibited platelet aggregation induced by arachidonic acid (AA) and platelet-activating factor (PAF). The inhibition (%) at 100 µg/mL was 96.2, and the anti-AA and anti-PAF IC_50_ (µg/mL) values were 11.5 and 58.6, respectively [139].

## 5. Conclusions

The *p*-coumaroyl amides identified in plants have many physiological protective roles, including antifeedant activity, cell wall fortification, and NO induction. The concentrations of these compounds are also higher in plants affected by several diseases. Different pathogens cause an increase in the levels of *p*-coumaroyl amides as well as an increase in the level of expression of the enzymes involved in their biosynthesis. The relevant roles of this class of compounds in plant self-protection have led to several attempts to modify genetically plants of agricultural interest in order to improve resistance to infections; however, as compounds such as *p*-coumaroylagmatine are widely available, their direct use for treating plants could be proposed.

Table 1 summarizes the most significant quantified results of their potential biological activities in humans and animals.

Their antioxidant activity (almost always measured) is perhaps the most important property as it is strongly associated with anti-inflammatory activities involving actions on many biochemical processes leading to inflammation and cardiovascular risk. Their anticancer properties have been studied to a smaller extent, limited to several specific cases. Conversely, their antidiabetic potential has been demonstrated in more examples. *p*-Coumaroyl amides have also been evaluated in neurological disorders as inhibitors of enzymes such as acetylcholine esterase, or as agonists/antagonists of synaptic receptors and modulators of neurotransmitter reuptake.

They have also been considered as antiviral in the inhibition of HIV protease and as antibacterial and anthelmintic.

The resulting activities are, in general, modest, and there are no compounds in our survey that could be directly considered potential drugs of the future. However, their beneficial effects in food and as food supplements are clearly demonstrated. Moreover, they could be interesting starting materials for developing semisynthetic bioactive compounds with enhanced activity due to their wide distribution in the plant kingdom, and the occurrence of many agricultural and food wastes that contain *p*-coumaroyl amides.

Compound **34** *N*^1^,*N*^5^,*N*^10^-tri-*p*-coumaroylspermidine showed the widest spectrum of biological activities, as demonstrated by the large number of publications reported in the literature.

## Figures and Tables

**Table 1:** Most relevant biological activities.

Compound	Plant Source	Enzyme/System	Activity	Comments	Reference
*p*-coumaroyltyrosine methyl ester **12**	*Hedera Rhombea*	α-Glucosidase inhibition	82.73 μM ^a^	One order of magnitude better than the reference inhibitor acarbose.	[113]
Abrusamide A **15**, Abrusamide D and H		NO synthase inhibition	23.4, 25.2 and 28.3 μM ^a^	In lipopolysaccharide (LPS)-induced RAW264.7 macrophages.	[27]
Feruloylserotonin and *p*-coumaroylserotonin **16**		Tyrosinase inhibition	23 and 74 μM ^a^	Notably, the melanogenesis inhibition test has also been carried out on human enzymes in melanoma cells	[30]
(*E,E*)-di-*p*-coumaroylputrescine **24**		Tyrosinase inhibition	181.73 µM ^a^		[45]
*p*-coumaroylserotonin **16**		Inhibition of and MAO-B AChE	MAO-B 1.2 μM AChE 15 μg/mL ^a^	MAO-B inhibition is potentially involved in AD development. AChE in scopolamine-induced mice to ameliorate memory	[33]
*N*^1^,*N*^5^-dicoumaroylspermidine **32** *N*^5^,*N*^10^-di-*p*-coumaroylspermidine **33** *N*^1^,*N*^5^,*N*^10^-tri-*p*-coumaroylspermidine **34**		AChE inhibition	714.43 μg/mL ^a^	Evaluation of an extract from foxtail millet bran	[60]
oleraciamide E **65**		AChE inhibition	52.43 ± 0.33 μM		[82]
*N*^10^-coumaroylspermidine **30**		Invertebrate neuromuscular junction receptors	200 μM ^a^	Antagonist action	[122]
Compound **55**		Inhibition of AchE and CarE	16.87 ± 0.83 and 23.14 ± 1.54 μM ^d^		[76]
Compound **56**		Inhibition of AchE and CarE	41.29 ± 1.54 and 46.87 ± 0.83 μM ^d^		[76]
(*Z*)-*N*^1^,*N*^5^-(*E*)-*N*^10^-tri-*p*-coumaroylspermidine **38**		Inhibition of serotonin uptake in S6 cells and in synaptosomes	0.74 ± 0.15 µM for S6 cells 1.07 ± 0.23 µM for synaptosomes ^a^		[63]
*N*^1^,*N*^5^,*N*^10^-tri-*p*-coumaroylspermidine **34**		HIV protease inhibition	83 μM ^a^		[44]
ailanthamide **72**		MLP/CB-induced elastase release	4.23 µg/mL ^a^		[134]
*N*^1^,*N*^5^-dicoumaroylspermidine **32**		Growth of HepG-2, MCF-7, and CaCo-2 cancer cell lines	46.34 ± 3.99 and 92.69 ± 5.63 μg/ml ^a^		[107]
Piperlotine E **81**		Platelet aggregation induced by arachidonic acid and PAF	11.5 µg/mL as anti-AA and 58.65 µg/mL as anti-PAF ^a^		[139]
Compound **45**		inhibitory activity on contraction of smooth muscle in guinea pig ileum and antagonism towards NK1 receptor	21.9 nM and 3.3 nM ^b^		[67]
*p*-Coumaroyloctopamine **54**		Antimicrobial activity against *S. aureus* and *K. pneumoniae*	64 mg/mL ^c^		[128]
Compound **27**		anthelmintic activity against *Dactylogyrus intermedius*	1.4 (mg/L) ^d^		[127]

^a^ IC_50_ (Half maximal inhibitory concentration); ^b^ K_i_ (Inhibition constant); ^c^ MIC (Minimum inhibitory concentration); ^d^ EC_50_ (Half maximal effective concentration).

## Data Availability

No new data were created.
